# Renewable Furfural-Based Polyesters Bearing Sulfur-Bridged Difuran Moieties with High Oxygen Barrier Properties

**DOI:** 10.1021/acs.biomac.2c00097

**Published:** 2022-03-23

**Authors:** Asmaa M. Ahmed, Tuomo P. Kainulainen, Juho Antti Sirviö, Juha P. Heiskanen

**Affiliations:** † Research Unit of Sustainable Chemistry, University of Oulu, P.O. Box 4300, FI-90014 Oulu, Finland; ‡ Fibre and Particle Engineering Research Unit, University of Oulu, P.O. Box 4300, FI-90014 Oulu, Finland

## Abstract

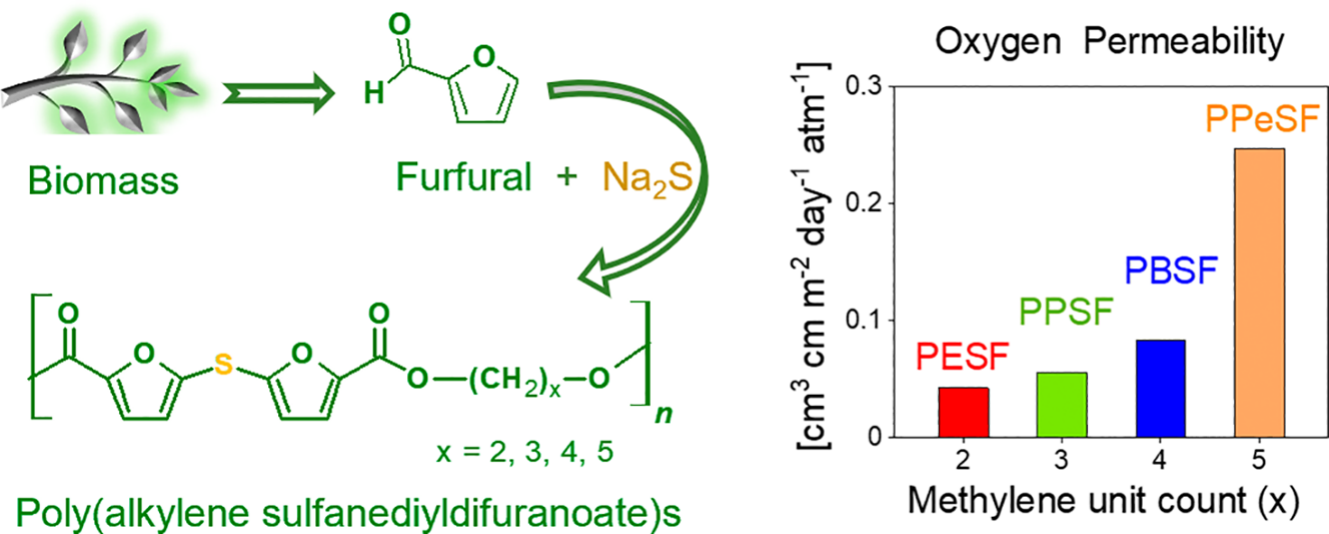

With the goal of achieving high barrier with bio-based materials, for example, for packaging applications, a series of novel furfural-based polyesters bearing sulfide-bridged difuran dicarboxylic acid units with high oxygen barrier properties were synthesized and characterized. For the novel poly(alkylene sulfanediyldifuranoate)s, a 11.2–1.9× higher barrier improvement factor compared to amorphous poly(ethylene terephthalate) was observed which places the novel polyesters in the top class among previously reported 2,5-furandicarboxylic acid (FDCA) and 2,2′-bifuran-based polyesters. Titanium-catalyzed polycondensation reactions between the novel synthesized monomer, dimethyl 5,5′-sulfanediyldi(furan-2-carboxylate), and four different diols, ethylene glycol, 1,3-propanediol, 1,4-butanediol, and 1,5-pentanediol, afforded difuran polyesters with high intrinsic viscosities (0.76–0.90 dL/g). These polyesters had good thermal stability, decomposing at 342–363 and 328–570 °C under nitrogen and air, respectively, which allowed processing them into free-standing films via melt-pressing. In tensile testing of the film specimens, tensile moduli in the range of 0.4–2.6 GPa were recorded, with higher values observed for the polyesters with shorter diol units. Interestingly, besides the low oxygen permeability, the renewable sulfide-bridged furan monomer also endowed the polyesters with slight UV shielding effect, with cutoff wavelengths of ca. 350 nm, in contrast to FDCA-based polyesters, which lack significant UV light absorption at over 300 nm.

## Introduction

Renewable resource-based feedstock chemicals and polymeric materials have recently attracted considerable attention owing to awareness about the progressive consumption of fossil resources, along with concerns about the environment. Furans are considered to be promising feedstocks for renewable materials. The chemistry of furan heterocycles has been the subject of extensive research because their numerous derivatives have valuable applications. Among possible furan green-platform compounds, 2-furaldehyde (furfural) and 5-hydroxymethylfurfural (HMF) constitute the two main dehydration products generated from pentoses and hexoses, respectively. These two basic bio-based furan derivatives find important uses as chemical precursors in a variety of industrial and fine chemical processes.^1−6^ The most significant example of a promising furan derivative is 2,5-furandicarboxylic acid (FDCA); it can be produced from direct oxidation of hydroxymethylfurfural or by multistep catalytic reactions of furfural.^7^ FDCA is listed in a 2004 US Department of Energy National Renewable Energy Laboratory report as one of the 12 building blocks that can be subsequently converted to a variety of high-value bio-based chemicals and polymeric materials.^8^ The polymerization of FDCA with ethylene glycol affords poly(ethylene furanoate) (PEF),^9−12^ which is often considered the most promising bio-based alternative to poly(ethylene terephthalate) (PET), a commodity polyester with a variety of useful properties.^13^ This is because the heteroaromatic FDCA moiety has certain similarities to the aromatic backbone moiety of PET, terephthalic acid.^14^ In addition to ethylene glycol, a wide range of flexible longer aliphatic glycols have been used to prepare various FDCA-based polyesters: poly(propylene furanoate) (PPF),^15^ poly(butylene furanoate) (PBF),^16,17^ poly(pentamethylene furanoate) (PPeF),^18^ and other poly(alkylene furanoate)s.^19,20^ They are typically synthesized via a polycondensation reaction between FDCA and the diol.

Furan-based polymers are interesting as they do not rely only on renewability but they instead also leverage superior performance comparable to commercial terephthalate analogues. For instance, in terms of gas permeability, O_2_ and CO_2_ transmission levels of PEF are significantly lowered by about 5.5–11× and 13–19×, respectively, compared to PET (Table 1).^10,11^ This excellent gas permeability reduction is attributed to the unique structure and polar character of furan ring reducing its segment mobility and hindering ring flipping.^21^ Moreover, a recently published study shows that FDCA-based homopolymers containing an odd count of methylene units, that is, PPF and PPeF, have superior gas barrier properties compared to PEF. When compared against PET, the barrier improvement factors (BIFs) of PPF and PPeF for oxygen were 16 and 227, respectively, and for carbon dioxide were 48 and 979, respectively. The exceptional barrier performance of these polyesters was attributed to the structural arrangement that allows the formation of 2D-ordered phase, mesophase.^18^

**Table 1:**
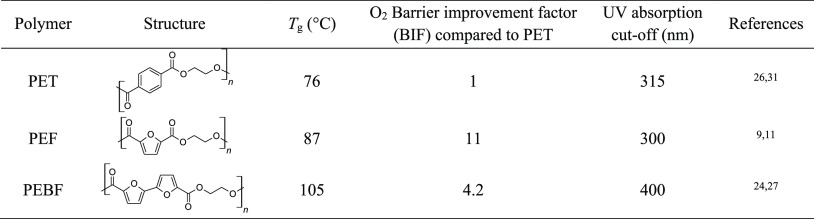
Selected Functional Properties of Bio-Based Polyesters with Different Building Blocks as Alternatives to Fossil-Based Polyester PET

Regarding applications of furfural in similar materials, attention has been focused on the study of 2,2′-bifuran-5,5′-dicarboxylic acid (BFDCA), the bifuran homologue of FDCA. BFDCA has been utilized as a monomer to synthesize novel bio-based polymers such as polyamides,^22^ polyesters,^23−29^ and resins.^30^ Interestingly, BFDCA-based poly(ethylene bifuranoate) (PEBF) has higher glass-transition temperature (*T*_g_) than its counterparts PET^31^ and PEF polymers (Table 1).^24^ Moreover, PEBF as well as other BFDCA-based polyesters may not only have good gas barrier performance but also intrinsic UV screening up to 400 nm.^24−26^

In view of all that has been mentioned so far, more contributions for the exploitation of specific features related to furan chemistry and effective utilization of furfural feedstock with the aim of synthesizing high-performance polymeric materials are required. The current study highlights a new furfural-derived monomer, which is used to prepare new bio-based furan polyesters containing the C–S–C bridge. At the beginning, the sulfur heteroatom is introduced between two units of furfural, yielding 5,5′-sulfanediyldi(furan-2-carbaldehyde). This compound has been utilized as a monomer for the production of polymers, for example, poly(Schiff base)s^32^ and polychalcones.^33^ However, polyesters containing that sulfur-bridged difuran moiety have not been utilized, characterized, or disclosed before, to the best of our knowledge. In this study, synthesis of the requisite monomer dimethyl 5,5′-sulfanediyldi(furan-2-carboxylate) is described, along with its use in polyesters. With the four different alkyl diols used in this study, a new family of renewable polyesters is introduced and evaluated in terms of material properties. It is found that the oxygen barrier performance of the polyesters is especially promising and that the polymers are mostly amorphous in character. Curiously, semicrystalline polyester was afforded with 1,3-propanediol, whereas ethylene glycol, 1,4-butanediol, and 1,5-pentanediol gave amorphous polyesters. Mechanically, they ranged from rigid and brittle to soft and ductile.

## Experimental Section

Diols, ethylene glycol (99.8%), 1,3-propanediol (98%), 1,4-butanediol (>99%), and 1,5-pentanediol (97%), were either purchased dry or distilled and stored under dry argon before use. Otherwise, commercially available chemicals and solvents were used as received. Sodium sulfide hydrate (>60% Na_2_S) was used as a source of sulfur in the reactions. Tetrabutyl titanate (TBT, 99%) was used as a catalyst for esterification and polycondensation reactions. Thin-layer chromatography was used for monitoring reactions using ethyl acetate/hexane (1:1) mixture as an eluent. CDCl_3_, (CD_3_)_2_SO, and CF_3_COOD were used for ^1^H and ^13^C NMR measurements.

### Syntheses

#### 5,5′-Sulfanediyldi(furan-2-carbaldehyde) (**1**)

A mixture of 5-bromofurfural (>98%, 10.71 g, 60 mmol), sodium sulfide hydrate (0.55 equiv, 4.29 g), and deionized water (200 mL) was heated at 95 °C for 2 h. The reaction mixture was allowed to cool down, and the precipitated powder was filtered, dried, and then purified by dissolving in dichloromethane followed by filtration through silica layer to afford a yellow-orange powder (5.33 g, 80%). Melting point: 130 °C. ^1^H NMR (400 MHz, CDCl_3_, ppm): δ 9.62 (s, 2H), 7.25 (d, 2H, *J* = 3.5 Hz), 6.81 (d, 2H, *J* = 3.5 Hz). ^13^C NMR (100 MHz, CDCl_3_, ppm): δ 177.3, 155.0, 147.7, 121.5, 118.7. NMR data agreed with previously reported data.^32,33^ HRMS (*m*/*z*): calcd for C_10_H_6_O_4_SNa [M + Na]^+^, 244.9879; found, 244.9877.

#### 5,5′-Sulfanediyldi(furan-2-carboxylic acid) (**2**)

5,5′-Sulfanediyldi(furan-2-carbaldehyde) (11.99 g, 54 mmol) and triethylamine (40 mL) were mixed in a 250 mL two-necked flask equipped with a magnetic stirring bar and a thermometer and placed in an ice bath. Once the reaction mixture temperature reached 0 °C, 30% hydrogen peroxide (4 equiv, 22 mL) was added to the reaction flask dropwise while keeping the temperature below 20 °C. After the addition of H_2_O_2_, the reaction flask was removed from the ice bath, and the mixture was stirred at room temperature for 2 h. Excess triethylamine was first recovered by distillation under reduced pressure, and then the reaction mixture was diluted with 500 mL of deionized water and acidified (pH 1–2) with 37% hydrochloric acid. The precipitated beige powder was filtered, washed with water, and dried. The crude product was purified by washing with warm ethanol followed by filtration and drying at room temperature to afford the pure product (13.03 g, 95%). ^1^H NMR (400 MHz, (CD_3_)_2_SO, ppm): δ 13.44 (br s, 2H), 7.27 (d, 2H, *J* = 3.4 Hz), 7.01 (d, 2H, *J* = 3.4 Hz). ^13^C NMR (100 MHz, (CD_3_)_2_SO, ppm): δ 159.0, 148.2, 145.1, 119.7, 119.6. HRMS (*m*/*z*): calcd for C_10_H_7_O_6_S [M + H]^+^, 254.9957; found, 254.9958.

#### Dimethyl 5,5′-Sulfanediyldi(furan-2-carboxylate) (**3**)

The reaction mixture of diacid compound **2** (5.08 g, 20 mmol) dissolved in dry methanol (150 mL) and concentrated sulfuric acid (2 equiv, 2.23 mL) was refluxed overnight at 65 °C. The yellow crystals of crude product formed during cooling were filtered, washed with cold methanol, and dried at room temperature. The product was collected as white crystals (5.21 g, 97%) after sublimation at 170 °C under a pressure of 0.1 mbar. Melting point: 153 °C. ^1^H NMR (400 MHz, CDCl_3_, ppm): δ 7.17 (d, 2H, *J* = 3.5 Hz), 6.74 (d, 2H, *J* = 3.5 Hz), 3.90 (s, 6H). ^13^C NMR (100 MHz, CDCl_3_, ppm): δ 158.3, 147.0, 146.0, 119.3. 118.5, 52.2. HRMS (*m*/*z*): calcd for C_12_H_11_O_6_S [M + H]^+^, 283.0270; found, 283.0271.

#### Poly(alkylene sulfanediyldifuranoate)s

TBT (50–400 ppm wt % Ti relative to monomer weight; 0.8–6.4 mg) was dissolved in dry toluene (0.5 mL) and mixed with the diol (3–5 equiv) along with monomer **3** (2.25 g, 8 mmol) in a 100 mL round-bottom flask equipped with a magnetic stirring bar. The flask was connected to a short-path distillation bridge and a receiving flask to collect the condensed methanol. The reaction system was evacuated and filled with argon gas for three cycles, and then the transesterification step was started by heating the mixture to 180 °C. The temperature was raised gradually to 200 °C over 2–4 h until no more methanol was received. For the polymerization step, the pressure was gradually reduced to 3 mbar over 1 h, after which the temperature was raised to 230–250 °C. Once the excess of diol was distilled off, the pressure was decreased to 0.16–0.11 mbar, and polymerization was continued for 3–5 h. The solid reaction product was dissolved in 20 mL of 1,1,1,3,3,3-hexafluoroisopropanol. The polyester was precipitated and washed using methanol and then filtered. Finally, the drying under vacuum at 60 °C afforded a white-to-off white fibrous polymer. The synthesis condition for each polymer is illustrated in Table 2. Polyester samples were dissolved in the mixture of CF_3_COOD/CDCl_3_ (1:3) for ^1^H NMR measurements and in CF_3_COOD for ^13^C NMR.

**Table 2:** Synthesis Condition and Results of Poly(alkylene sulfanediyldifuranoate)s

				polymerization			
polyester	diol equiv	catalyst amount (ppm Ti)	esterification time (h)	time (h)	temp (°C)	yield (%)	IV (dL/g)^a^
PESF	5	400	4	5	250	94	0.90
PPSF	3	50	3	5	250	91	0.86
PBSF	3	50	2	3	230	97	0.83
PPeSF	3	50	2	3	230	92	0.76

a IV at 30 °C in phenol/1,1,2,2-tetrachloroethane 60:40 w/w (*c* = 0.5 g/dL).

### Characterization

#### Dilute Solution Viscometry

Intrinsic viscosities (IVs) were determined from the flow times of phenol:1,1,2,2-tetrachloroethane (60:40, w/w) mixture (*t*_0_) and 0.5 g/dL polyester solutions (*t*) in a micro-Ubbelohde viscometer submerged in 30.0 °C water bath. The average of three flow times was used to calculate *t*_0_ and *t*. Billmeyer equation was used to calculate the IV according to the ASTM D 4603 technique.^34^

#### Fourier Transform Infrared

Fourier transform infrared (FTIR) spectra were collected in the wavenumber range of 600–4000 cm^–1^ using attenuated total reflectance technique (PerkinElmer Spectrum 100 equipped with a Specac ATR accessory). 16 scans were collected at a resolution of 2 cm^–1^ at room temperature.

#### Differential Scanning Calorimetry

Differential scanning calorimetry (DSC) (Mettler Toledo DSC 821e) was used to investigate the melting point of synthesized products and the thermal behavior of polyesters in the range of −10 to 250 °C under N_2_ gas flow (50 cm^3^/min) with heating and cooling rates of 10 °C/min.

#### Thermogravimetric Analysis

Thermogravimetric analyzer (TGA) (STA409P) was used to evaluate thermal stability and decomposition properties of polymers in the heating range of 37–700 °C under an air or pure nitrogen atmosphere using the flow rate of 40 cm^3^/min and a heating rate of 10 °C/min.

#### Film Processing

Films of 100–200 μm thickness were prepared by melt-press, starting from the dried fibrous homopolymers, by means of a Fontijne Hydraulic Press (LabEcon 300). The appropriate amount of carefully dried polyester (1.5 g) was placed between two preheated aluminum plates covered with smooth polytetrafluoroethylene-coated glass fiber sheets. The thickness of pressed films was controlled using layers of aluminum frame placed around the plates. The polyesters were melted at temperatures 20–30 °C above their expected melting temperatures for a few minutes to absolute melting and then pressed for 2 min at 30 and 50 kN. The water-cooling circuit of the press was used to cool down the plates. Then, the aluminum plates were separated, and the films were peeled off carefully to yield flexible, transparent, low-crystalline films. Commercial PET pellets were used to prepare amorphous PET reference films. It is worth noticing that the process conditions did not affect the chemical structure of the materials, validated by NMR measurements of the melt-pressed film samples, and the produced films were suitable for the characterization of thermomechanical properties and barrier performance.

#### Polyester Film Thickness

A thickness gauge (precision thickness Gauge FT3, Hanatek Instrument, UK) was used to measure the exact thickness of polymer films and specimens used for characterization.

#### Tensile Testing

Rectangle-shaped specimens of polyester films with 5 mm width and thicknesses between 100 and 200 μm were stored under stable conditions (23 °C, 50% RH) for 48 h before the tests. Tensile testing (Instron 5544, USA) was performed under the same conditions using a gauge length of 30 mm and a crosshead speed of 5 mm/min. The reported mechanical results were obtained from five measurements carried out on each polymer.

#### Dynamic Mechanical Analysis

The dynamic mechanical properties of the prepared polyesters were evaluated from rectangular-shaped melt-pressed film pieces using dynamic mechanical analysis (DMA, Q800, TA Instruments, USA). All runs were performed in a “multi-frequency, strain” mode at 1 Hz, 0.08% strain at a heating rate of 3 °C/min.

#### Gas Permeability Analysis

The oxygen permeability (OP) of the melt-pressed polyester films as well as reference PET films was measured using MOCON OxTran 2/20 at 23 °C and 0% RH (relative humidity) with a specimen exposure area of 5 cm^2^. OP values were measured twice from duplicate samples of melt-pressed films with thicknesses of 100–200 μm. The value of BIF was calculated based on the OP of reference PET films divided by that of polyester films.

#### UV–Vis

A Shimadzu UV-1800 spectrophotometer was used to acquire transmission spectra of thin rectangle-shaped film pieces within the wavelength of 200–800 nm.

## Results and Discussion

### Monomer Synthesis

**Scheme 1:**
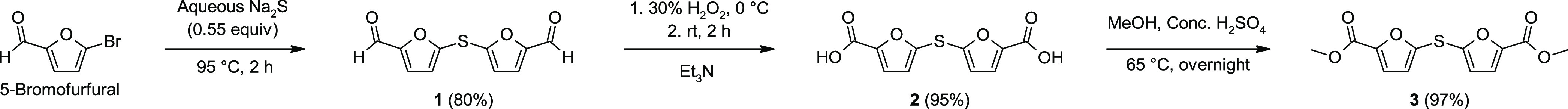
Synthesis of Dimethyl 5,5′-Sulfanediyldi(furan-2-carboxylate) (**3**)

The target monomer, dimethyl 5,5′-sulfanediyldi(furan-2-carboxylate) (**3**), was synthesized over three reaction steps starting from 5-bromofurfural (Scheme 1): building the sulfur linkage, oxidation of the dialdehyde, and esterification. The presence of the halogen atom in the 5 position of the furan ring facilitates its replacement possibilities by nucleophiles such as sulfide. Thus, the reaction of 5-bromofurfural with sodium sulfide afforded dialdehyde **1** in 80% yield. Oxidation of the resulting dialdehyde was somewhat problematic since electron-rich furfural derivatives tend to favor Dakin oxidation-type products when reacted with hydroperoxides.^35−37^ However, in the presence of triethylamine, these side reactions are suppressed, and aqueous 30% hydrogen peroxide becomes an effective oxidant. These conditions appeared completely selective toward the aldehyde groups, that is, sulfur was not oxidized. Even heating (to ca. 60 °C) did not appear to result in oxidation of sulfur. The resulting dicarboxylic acid (**2**) was finally esterified with refluxing dry methanol and sulfuric acid as a catalyst, yielding dimethyl ester (**3**). Overall, the developed method offers a simple and transition metal-free synthetic protocol to turn a monofunctional furfural unit into a difunctional monomer. The chemical structures of the synthesized compounds were validated by NMR, FTIR, and HRMS analyses (Figures S1–S3 in the Supporting Information).

### Polyester Synthesis

**Scheme 2:**
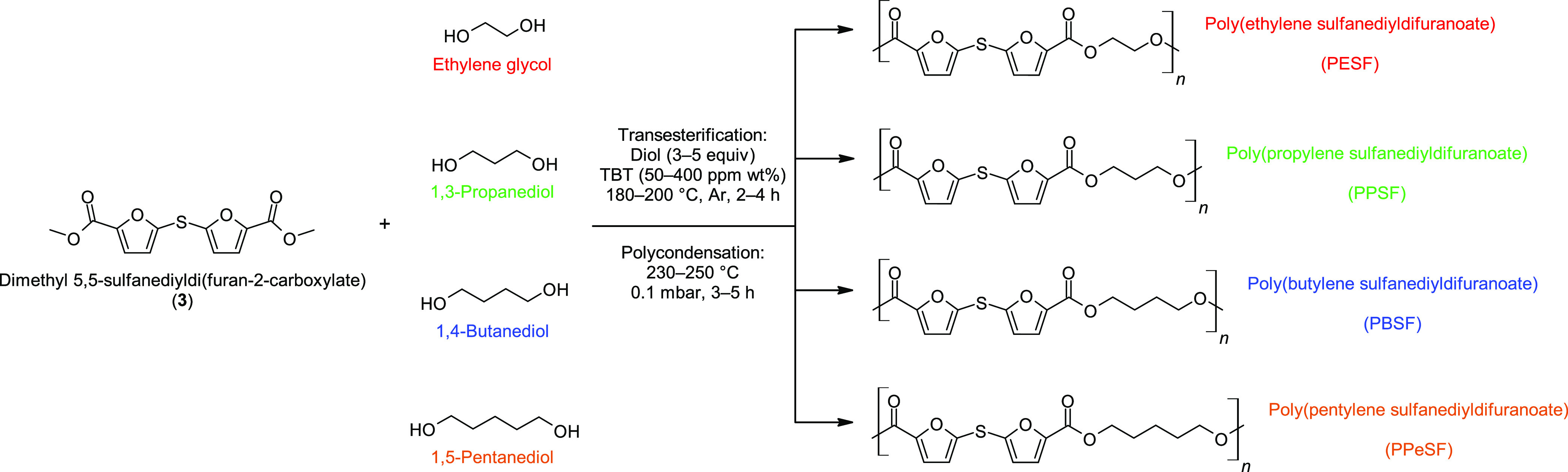
Synthesis of Polyesters: PESF, PPSF, PBSF, and PPeSF

With the successful preparation of the monomer, a series of high-molecular-weight polyesters were synthesized with modest levels of titanium catalyst. A higher amount of catalyst was employed with ethylene glycol to offset its lower reactivity and to ensure a high molecular weight. The two-stage melt polycondensation procedure, transesterification and polymerization, was employed to synthesize poly(alkylene sulfanediyldifuranoate)s (Scheme 2). In the first step, monomer **3** was transesterified with diol in the presence of TBT under an argon atmosphere to yield the dihydroxyl ester intermediate. By the end of the esterification step, the reaction pressure was reduced gently to remove the excess diol. The intermediate oligomers were polymerized in the second step under high temperature and low pressure to afford polyesters, PESF, PPSF, PBSF, and PPeSF (Scheme 2), in high yields (Table 2). The IVs of the prepared polyesters ranged between 0.76 and 0.90 dL/g confirming the high molecular weight of the polyesters and being in the range of that corresponding to the IV of PET quality used in fiber and bottle applications.^13^

**Figure 1:**
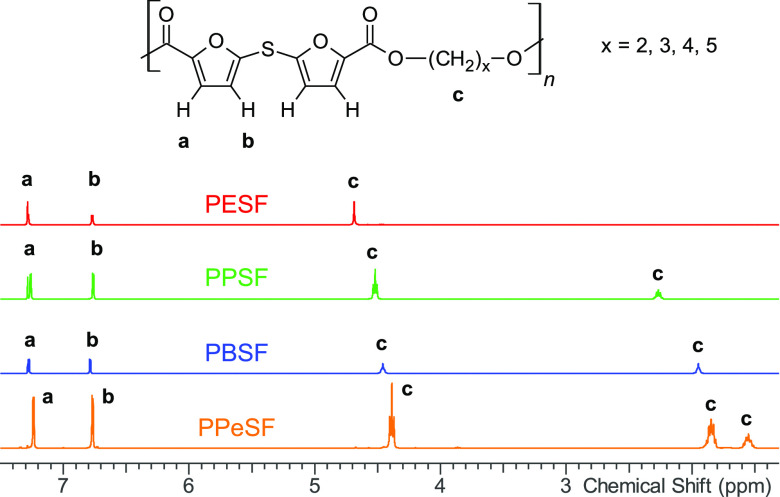
^1^H NMR signal assignments of poly(alkylene sulfanediyldifuranoate)s.

### Polyester Characterization

The structures of the synthesized polyesters were confirmed by ^1^H and ^13^C NMR measurements (Figures S4–S7). In all ^1^H NMR spectra of the synthesized polyesters (Figure 1), the two sets of doublets (a and b) in the range of 6.5–7.5 ppm are ascribed to the protons of the furan ring, while alkyl protons (c) are detected in the chemical shift range of 1.4–4.7 ppm. Similarly, ^13^C NMR spectra of all polyesters bear the signals of furan ring carbons from 120 to 150 ppm as well as the carbonyl group at around 163 ppm. The alkyl chain carbons are detected in the range of 70–23 ppm.

In the same vein, FTIR spectra of the polyester series demonstrated notable similarity to each other (Figure S8). The characteristic adsorption peaks of the 2,5-disubstituted furan ring are observed for all samples, including the symmetric and asymmetric stretching vibrations of C–H (3148 and 3123 cm^–1^), C=C (1579 cm^–1^), the furan ring breathing vibration (1015 cm^–1^), and the ring bending vibrations (935, 812, and 755 cm^–1^).^38^ The ester bond stretching vibrations were indicated by peaks of C=O at 1714 cm^–1^ and C–O–C at 1283 cm^–1^. A weak vibration peak of C–S–C was observed at 673 cm^–1^. With increasing alkyl chain, the asymmetric and symmetric stretching vibration intensities of C–H bonds at 2924–2961 and 2855–2896 cm^–1^, respectively, increase. In addition, the FTIR results imply that the analyzed polyester samples have high molecular weight since the possible terminal hydroxyl group peak around 3400 cm^–1^ was not detected in any polyester spectra.

### Thermal Properties

**Figure 2:**
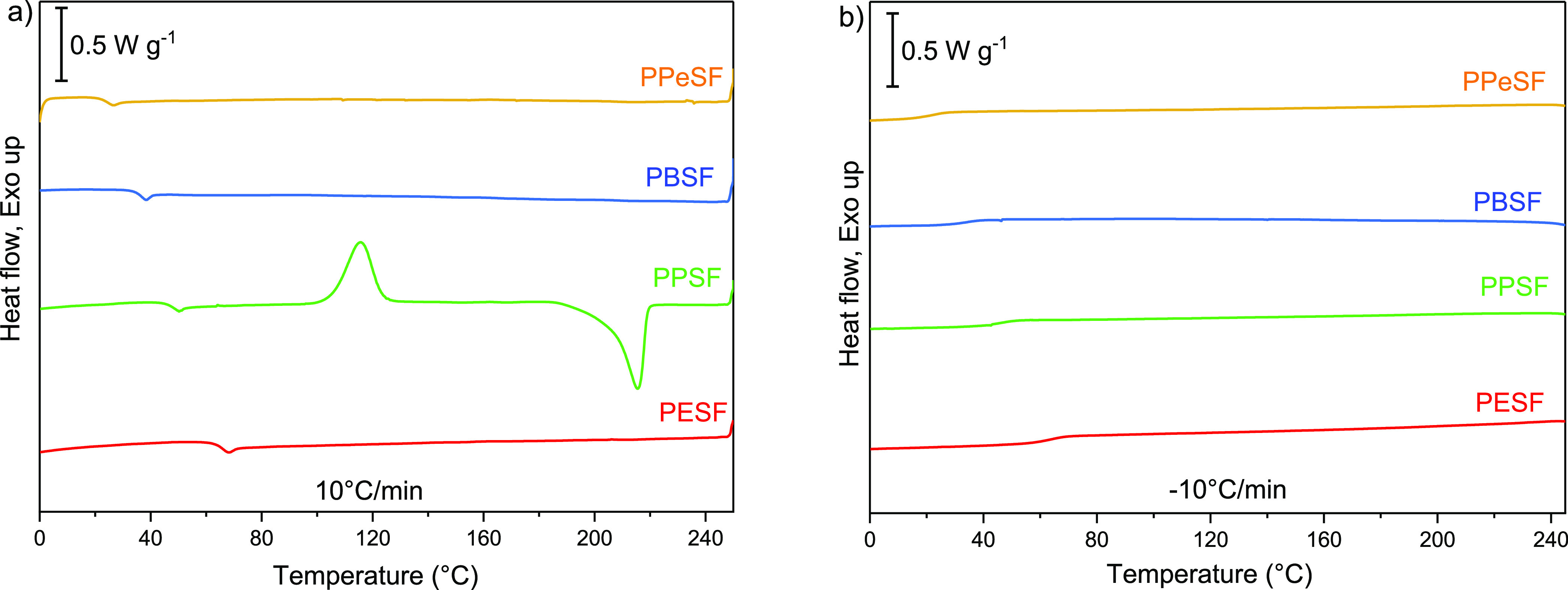
DSC thermograms of polyesters. (a) Second heating and (b) second cooling.

The thermal characteristics of the polyesters are presented in Figure 2 and Table 3. The correlation between the glass transition temperature and the alkyl chain length is noted. The presence of flexible groups like the aliphatic chains tends to decrease the *T*_g_ as a result of reducing polymer chain rigidity and hence increases the chain mobility and free volume.^39^ Thus, PPeSF showed the lowest *T*_g_ value at 26 °C, while PESF demonstrated the highest at 65 °C. Except for PPSF, neither exotherms nor endotherms, from crystallization or melting, respectively, were observed in the second heating scans of any of the polyesters, which is due to the amorphous character of these materials. In contrast to other polyesters, PPSF showed exothermic crystallization peak at 118 °C in both heating scans, revealing its semicrystalline structure. The observed multiple melting peaks of PBSF (Figure S9) had been attributed to the presence of different crystal morphologies resulting from solvent crystallization as polyester samples were precipitated from solution into methanol and dried at 60 °C for several days. Similar multiple melting endotherms were reported in furan-based polyesters, for example, PEF.^14,40^

**Table 3:** Thermal Properties of Poly(alkylene sulfanediyldifuranoate)s^a^

	DSC				TGA			
					N_2_		air	
sample	*T*_g_ (°C)	*T*_m_ (°C)	*T*_cc_ (°C)	*T*_d5_ (°C)	*T*_d50_ (°C)	R_700_ (%)	*T*_d5_ (°C)	*T*_d50_ (°C)
PESF	65	164	nd	363	383	23.2	352	376
PPSF	45	216	118	345	384	23.3	340	387
PBSF	37	104 & 134	nd	348	381	19	334	388
PPeSF	26	85	nd	342	381	15.8	328	384

a *T*_g_: glass transition temperature from second cooling. *T*_m_: melting temperature from first heating. *T*_cc_: cold crystallization peak from first heating. *T*_d5_: temperature at 5% sample mass-loss. *T*_d50_: temperature at 50% sample mass-loss. *R*_700_: residual mass at 700 °C. nd: not detected.

**Figure 3:**
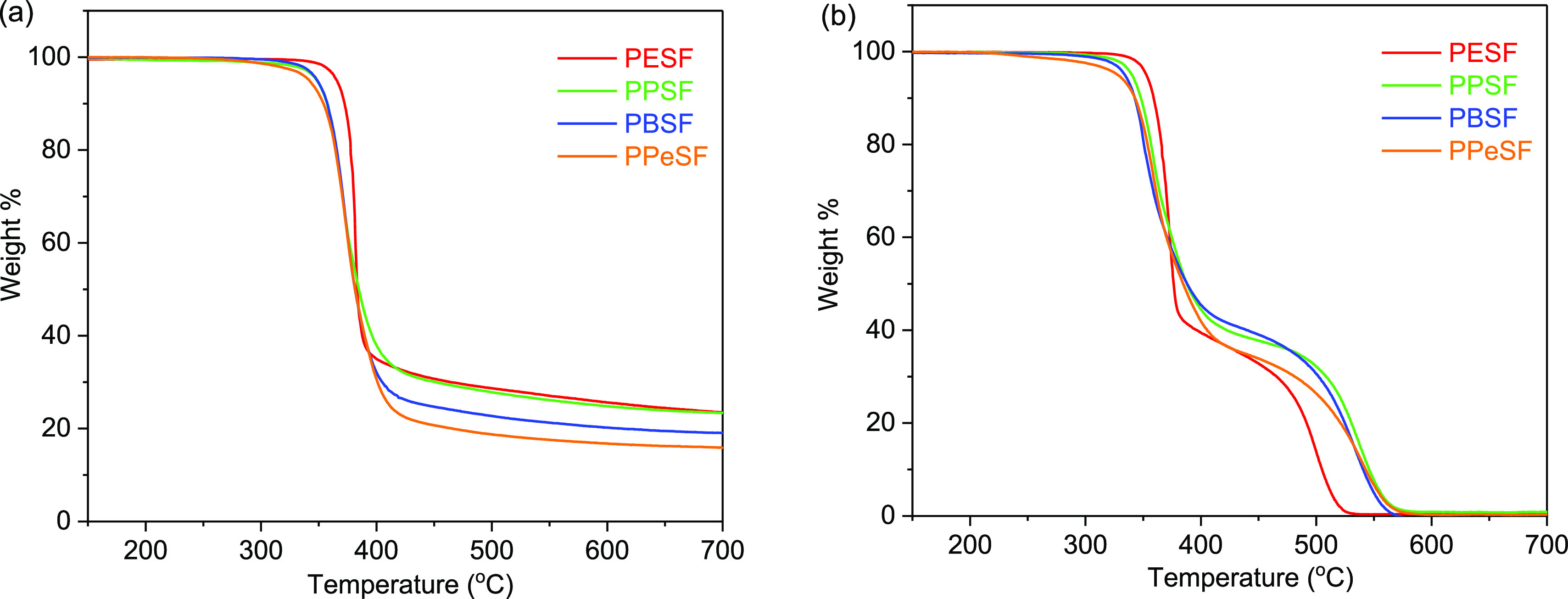
Thermogravimetric decomposition curves for the synthesized polyesters under (a) nitrogen atmosphere and (b) air atmosphere.

Thermal stabilities of the polyesters were analyzed by comparing the decomposition temperatures at which the weight loss reached 5% of the initial weight (*T*_d5_) and those at 50% of the initial weight (*T*_d50_) (Table 3). These characteristics were measured under both air and nitrogen atmospheres with a heating rate of 10 °C/min, as shown in Figure 3. Thermal stabilities as well as residual masses depended on the alkyl chain length of the diol used as the monomer. Thermal stability and char yield therefore decreased gradually from PESF to PPeSF. Under a N_2_ atmosphere (Figure 3a), the polyesters decomposed in one step starting from 342 °C, with complete decomposition at ca. 380 °C. Under air, the rate of decomposition was somewhat faster relative to that under nitrogen, and samples were degraded in the two-stage process (Figure 3b). During the two-stage decomposition, the polymers were first degraded at 328–352 °C in the major step, while the resultant char residue was burned off in the minor step. In the minor decomposition step, PESF showed faster mass-loss rate and was completely consumed at 530 °C compared to other polymers at around 570 °C. In conclusion, thermal stabilities of the synthesized polyesters fall in the same range as that corresponding to FDCA-based polyesters, that is, PEF, PPF, and so forth.^41^

### Mechanical Properties

**Figure 4:**
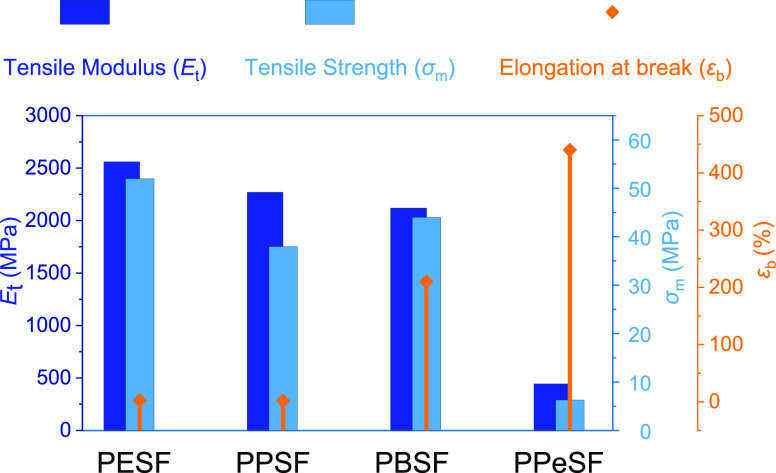
Mechanical characterization data obtained by stress–strain measurements of poly(alkylene sulfanediyldifuranoate)s.

The mechanical properties of the novel poly(alkylene sulfanediyldifuranoate)s are highlighted in Table 4 and Figure 4. Except for PPeSF, all polyesters showed a comparably high tensile modulus (*E*_t_) ranging from 2119 to 2560 MPa and tensile strength (σ_m_) from 44 to 52 MPa. Elongation at break (ε_b_) ranged from 1.9 to 210%. The mechanical performance of the polymer is affected by several factors such as chain flexibility and degree of crystallinity. Particularly, the tensile elastic modulus and the tensile strength decrease, while the elongation at break increases as the alkyl chain length of the diol increases. This trend could be attributed to an increase in the chain mobility and a decrease in the glass transition temperature of polyesters. Similar behavior has been previously observed in FDCA-based polyesters, for example, PPF, PBF, and PPeF.^18^ PESF and PPSF showed the highest values of tensile modulus and tensile stress but low ductility. Far higher elongation (increase from 2% to over 200%) was observed for PBSF with otherwise comparable performance. In contrast, PPeSF had low tensile modulus and strength but even higher elongation at break (>400%). It obviously suffers from its low *T*_g_ that is very close to the temperature maintained during the tests. PPeSF shows the typical elastomeric response with the absence of yielding and almost complete recovery after elongation, as previously reported for PPeF, derived from FDCA.^42^ In conclusion, the results obtained from stress–strain measurements indicate that changing the diol subunit length represents an efficient tool to tune the mechanical response of polyesters derived from monomer **3**, providing stiff as well as flexible polymeric materials.

The viscoelastic properties of the polyesters were analyzed by DMA. The thermograms with storage modulus *E*′, loss modulus *E*″, and tan δ as a function of temperature for melt-pressed polyester films are presented in Figure S10. The *T*_g_ values evaluated from the peak of the tan δ or *E*″ curve agreed with DSC (Table S1). A notable feature in the DMA thermogram of PPSF is the appearance of *E*′ plateau due to cold crystallization, observed after about 95 °C, which is consistent with DSC. Accordingly, cold crystallization was not observed in the other polyesters.

**Table 4:** Measured Tensile Properties of the Film Specimens Prepared from Poly(alkylene sulfanediyldifuranoate)s

sample^a^	*E*_t_ (MPa)	σ_m_ (MPa)	ε_b_ (%)
PESF	2560 ± 300	52 ± 5	2.3 ± 0.5
PPSF	2270 ± 200	38 ± 8	1.9 ± 0.3
PBSF	2119 ± 120	44 ± 6	210 ± 53
PPeSF	445 ± 27	6.3 ± 0.5	440 ± 100

a At least five amorphous specimens were evaluated for each polyester. *E*_t_ = tensile modulus. σ_m_ = maximum tensile stress. ε_b_ = elongation at break.

### Oxygen Permeability

Developing polyesters with good barrier properties by controlling their chemical structure is vital for making materials marketable especially toward packaging applications. It is generally accepted that improving the protective function of food packaging extends shelf life and, consequently, reduces food losses, thus reducing the environmental impact. Therefore, high oxygen barrier packaging materials are required. In fact, furan-based polymers are already well-known for their low OP compared with PET. For these reasons, OP and BIF of poly(alkylene sulfanediyldifuranoate)s as well as PET (as a reference) were measured and calculated. The results are presented in Table 5 with the comparison to previous results of FDCA-based PEF, PPF, and PBF together with bifuran-based polyesters PEBF and PBBF to evaluate the oxygen barrier properties of the prepared novel polyesters.

OP of poly(alkylene sulfanediyldifuranoate)s showed that the permeability depended on the alkyl chain length, where the polymer with longer alkyl chain parts exhibited higher OP. PESF, PPSF, and PBSF had BIFs of 11.2×, 8.5×, and 5.7×, respectively, against the amorphous PET reference films. The barrier performance of PPeSF is notably lower relative to other novel polyesters, which may be in part because of its low *T*_g_ combined with the highest methylene unit count. Nevertheless, the oxygen barrier performance of PPeSF is still nearly 2 times better than PET. The results show clearly that the novel polyesters derived from monomer **3** are extremely effective for reducing O_2_ gas transmission, offering similar high barrier performance as FDCA-based polyesters PEF, PPF, PBF. The presence of the combined furan and sulfide motifs in the polymer chain is promising for the further development of improved biobased gas barrier polymers.

### UV-Screening Properties

**Figure 5:**
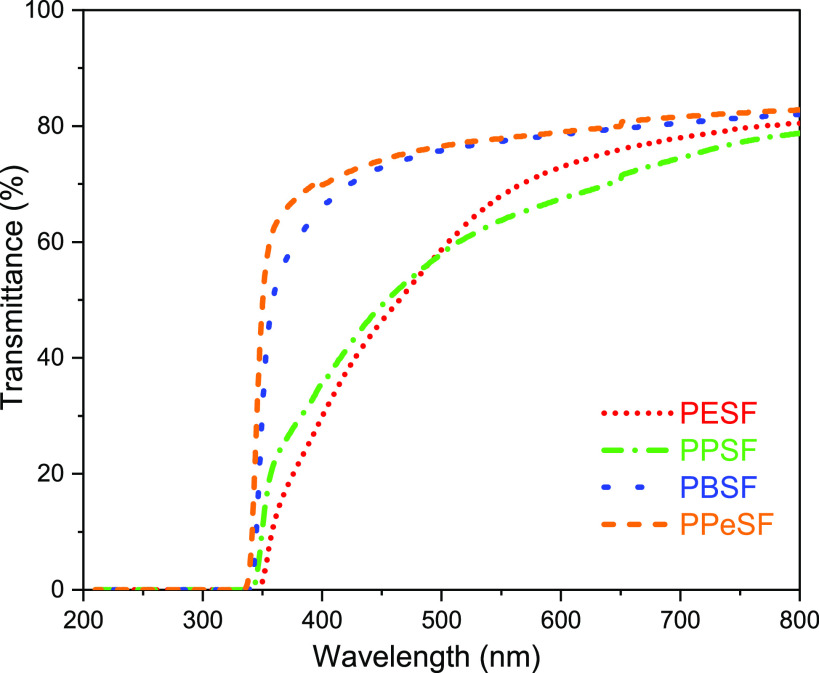
UV–vis transmittance curves of melt-pressed poly(alkylene sulfanediyldifuranoate)s films.

**Figure 6:**
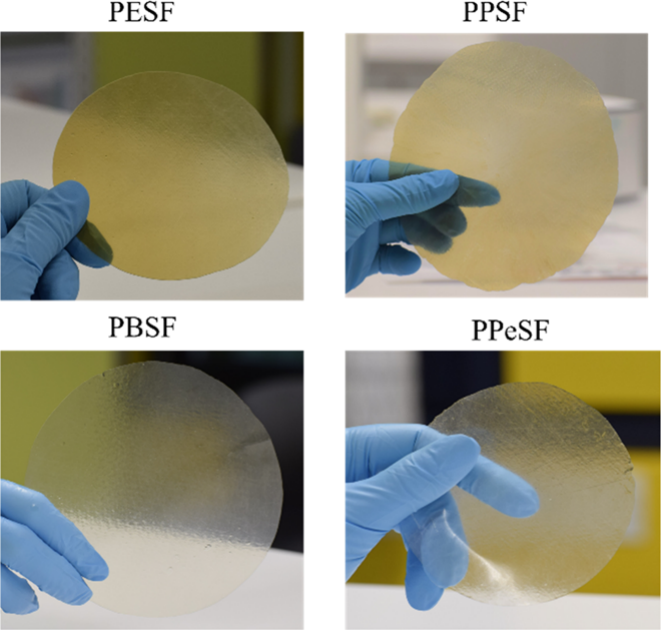
Digital image of melt-pressed poly(alkylene sulfanediyldifuranoate)s films.

UV-blocking ability of the films can be a desired characteristic in order to protect sensitive goods against UV radiation. At the same time, the transparency of polymer films is often considered an important property, for example, in packaging applications. As mentioned previously (Table 1), polyesters derived from BFDCA generally have a broad UV absorbance up to 400 nm due to extended conjugated system of the present bifuran unit, while FDCA-based polyesters lack significant UV light absorption at over 300 nm. The UV transmittances of poly(alkylene sulfanediyldifuranoate)s fall in the range between FDCA and BFDCA polyesters, providing good UV light filtering ability up to 350 nm wavelength (Figure 5). Clearly, the presence of a sulfur bridge between the two furan rings is capable of extending the conjugation of monomer **3** compared to FDCA, resulting in ca. 50 nm red-shifted absorption. Distinctly, the melt-pressed polyester films exhibited simultaneously good visual transparencies with light-yellow appearance (Figure 6). The observed yellowness of the produced polymer films has been recognized in the furan-based polymer literature previously.^43,44^ It seems logical, when comparing PESF and PPSF to PBSF and PPeSF, that the elevated temperatures and the extended reaction time result in more intensely colored side-products. TBT and similar titanium catalysts are also known to have a strong tendency to impart color.^45^ As a conclusion, the presence of sulfur bridge between two furan rings results in extended conjugation in monomer **3** which provides a low UV transmittance ability for the synthesized polyester films that is another essential property in packaging applications in addition to good gas barrier properties.

**Table 5:** O_2_ Barrier Properties of Poly(alkylene sulfanediyldifuranoate)s Films with Respect to Other Polyesters

sample	OP^a^	BIF^b^	conditions
PET	0.4737	1	current study at 23 °C, 0% RH
PESF	0.0422	11.2	
PPSF	0.0555	8.5	
PBSF	0.0827	5.7	
PPeSF	0.2472	1.9	
PEF^c^	0.0107	11	35 °C
PPF^d^	0.0472	8	23 °C, 50% RH
PBF^e^	0.162	4	25 °C, 50% RH
PEBF^f^	0.269	2.4	23 °C, 0% RH
PEBF^g^	0.110	4.2	23 °C, 0% RH
PBBF^e^	0.188	3.5	25 °C, 50% RH

a OP in [cm^3^ cm m^–2^ day^–1^ atm^–1^].

b Calculated with the formula: BIF = OP_(PET)_/OP_(Polyester)_. RH = relative humidity.

c References (9) and (11).

d Reference (15).

e Reference (25).

f Reference (24).

g Reference (27).

## Conclusions

The novel furfural-derived monomer, dimethyl 5,5′-sulfanediyldi(furan-2-carboxylate), containing the sulfur-bridged difuran moiety was synthesized and utilized in the preparation of series of novel furan-based polyesters with high oxygen barrier properties for the first time. The prepared poly(alkylene sulfanediyldifuranoate)s showed 11.2–1.9× higher BIF compared to amorphous PET. The reported findings place the novel furfural-derived polyesters in the top class among previously reported FDCA and 2,2′-bifuran-based polyesters. Glass transition temperatures of the different polyesters were in a relatively narrow range of 26–65 °C. Thermal stabilities corresponded quite closely to those of similar FDCA-derived polyesters, suggesting that the thermal stability was not affected much by the sulfur linkage. All polyesters showed amorphous character apart from PPSF, which appeared more prone to crystallize. Once melt-pressed, the polyesters were transparent in accordance with their amorphous character. Their cutoff wavelengths for transmission were in the UV region at ca. 350 nm, providing improved UV blocking ability compared to FDCA-based polyesters. The mechanical performance of the polyesters was comparable to other thermoplastic polyesters of similar nature. In conclusion, the developed method allows the effective utilization of biomass-derived platform chemical, furfural, as a precursor for high oxygen barrier polyester materials. The reported properties of these novel polyesters add further evidence to the fact that furan-derived polyesters are high-performance materials in many senses.

## Supporting Information Available

Characterization data; ^1^H and ^13^C NMR spectra; FTIR spectra of polyesters; DSC thermograms of the synthesized polyesters; DMA thermograms of melt-pressed polyester films; and storage modulus and *T*_g_ determined by DMA for the synthesized polyesters (PDF)

## References

[ref1] Bozell J. J.; Petersen G. R. Technology Development for the Production of Biobased Products from Biorefinery Carbohydrates—the US Department of Energy’s “Top 10” Revisited. Green Chem. 2010, 12, 539–555. 10.1039/b922014c.

[ref2] Zhang Q.; Song M.; Xu Y.; Wang W.; Wang Z.; Zhang L. Bio-Based Polyesters: Recent Progress and Future Prospects. Prog. Polym. Sci. 2021, 120, 101430 10.1016/j.progpolymsci.2021.101430.

[ref3] Mariscal R.; Maireles-Torres P.; Ojeda M.; Sádaba I.; López Granados M. Furfural: A Renewable and Versatile Platform Molecule for the Synthesis of Chemicals and Fuels. Energy Environ. Sci. 2016, 9, 1144–1189. 10.1039/c5ee02666k.

[ref4] Zhang D.; Dumont M.-J. Advances in Polymer Precursors and Bio-Based Polymers Synthesized from 5-Hydroxymethylfurfural. J. Polym. Sci., Part A: Polym. Chem. 2017, 55, 1478–1492. 10.1002/pola.28527.

[ref5] Gandini A. Furans as Offspring of Sugars and Polysaccharides and Progenitors of a Family of Remarkable Polymers: A Review of Recent Progress. Polym. Chem. 2010, 1, 245–251. 10.1039/b9py00233b.

[ref6] Zhao Y.; Lu K.; Xu H.; Zhu L.; Wang S. A Critical Review of Recent Advances in the Production of Furfural and 5-Hydroxymethylfurfural from Lignocellulosic Biomass through Homogeneous Catalytic Hydrothermal Conversion. Renew. Sustain. Energy Rev. 2021, 139, 110706 10.1016/j.rser.2021.110706.

[ref7] Zhang S.; Lan J.; Chen Z.; Yin G.; Li G. Catalytic Synthesis of 2,5-Furandicarboxylic Acid from Furoic Acid: Transformation from C5 Platform to C6 Derivatives in Biomass Utilizations. ACS Sustainable Chem. Eng. 2017, 5, 9360–9369. 10.1021/acssuschemeng.7b02396.

[ref8] Werpy T.; Petersen G. Top Value Added Chemicals from Biomass Volume I-Results of Screening for Potential Candidates from Sugars and Synthesis Gas; The Pacific Northwest National Laboratory (PNNL) and The National Renewable Energy Laboratory (NREL), 2004.

[ref9] Burgess S. K.; Leisen J. E.; Kraftschik B. E.; Mubarak C. R.; Kriegel R. M.; Koros W. J. Chain Mobility, Thermal, and Mechanical Properties of Poly(Ethylene Furanoate) Compared to Poly(Ethylene Terephthalate). Macromolecules 2014, 47, 1383–1391. 10.1021/ma5000199.

[ref10] Burgess S. K.; Kriegel R. M.; Koros W. J. Carbon Dioxide Sorption and Transport in Amorphous Poly(Ethylene Furanoate). Macromolecules 2015, 48, 2184–2193. 10.1021/acs.macromol.5b00333.

[ref11] Burgess S. K.; Karvan O.; Johnson J. R.; Kriegel R. M.; Koros W. J. Oxygen Sorption and Transport in Amorphous Poly(Ethylene Furanoate). Polymer 2014, 55, 4748–4756. 10.1016/j.polymer.2014.07.041.

[ref12] Araujo C. F.; Nolasco M. M.; Ribeiro-Claro P. J. A.; Rudić S.; Silvestre A. J. D.; Vaz P. D.; Sousa A. F. Inside PEF: Chain Conformation and Dynamics in Crystalline and Amorphous Domains. Macromolecules 2018, 51, 3515–3526. 10.1021/acs.macromol.8b00192.

[ref13] Ji L. N. Study on Preparation Process and Properties of Polyethylene Terephthalate (PET). Appl. Mech. Mater. 2013, 312, 406–410. 10.4028/www.scientific.net/AMM.312.406.

[ref14] Papageorgiou G. Z.; Tsanaktsis V.; Bikiaris D. N. Synthesis of Poly(Ethylene Furandicarboxylate) Polyester Using Monomers Derived from Renewable Resources: Thermal Behavior Comparison with PET and PEN. Phys. Chem. Chem. Phys. 2014, 16, 7946–7958. 10.1039/c4cp00518j. 24647534

[ref15] Vannini M.; Marchese P.; Celli A.; Lorenzetti C. Fully Biobased Poly(Propylene 2,5-Furandicarboxylate) for Packaging Applications: Excellent Barrier Properties as a Function of Crystallinity. Green Chem. 2015, 17, 4162–4166. 10.1039/c5gc00991j.

[ref16] Ma J.; Yu X.; Xu J.; Pang Y. Synthesis and Crystallinity of Poly(Butylene 2,5-Furandicarboxylate). Polymer 2012, 53, 4145–4151. 10.1016/j.polymer.2012.07.022.

[ref17] Bianchi E.; Soccio M.; Siracusa V.; Gazzano M.; Thiyagarajan S.; Lotti N. Poly(Butylene 2,4-Furanoate), an Added Member to the Class of Smart Furan-Based Polyesters for Sustainable Packaging: Structural Isomerism as a Key to Tune the Final Properties. ACS Sustain. Chem. Eng. 2021, 9, 11937–11949. 10.1021/acssuschemeng.1c04104. 34513341 PMC8424682

[ref18] Guidotti G.; Soccio M.; García-Gutiérrez M. C.; Ezquerra T.; Siracusa V.; Gutiérrez-Fernández E.; Munari A.; Lotti N. Fully Biobased Superpolymers of 2,5-Furandicarboxylic Acid with Different Functional Properties: From Rigid to Flexible, High Performant Packaging Materials. ACS Sustain. Chem. Eng. 2020, 8, 9558–9568. 10.1021/acssuschemeng.0c02840. 33796416 PMC8007128

[ref19] Jiang M.; Liu Q.; Zhang Q.; Ye C.; Zhou G. A Series of Furan-Aromatic Polyesters Synthesized via Direct Esterification Method Based on Renewable Resources. J. Polym. Sci., Part A: Polym. Chem. 2012, 50, 1026–1036. 10.1002/pola.25859.

[ref20] Terzopoulou Z.; Tsanaktsis V.; Nerantzaki M.; Achilias D. S.; Vaimakis T.; Papageorgiou G. Z.; Bikiaris D. N. Thermal Degradation of Biobased Polyesters: Kinetics and Decomposition Mechanism of Polyesters from 2,5-Furandicarboxylic Acid and Long-Chain Aliphatic Diols. J. Anal. Appl. Pyrolysis 2016, 117, 162–175. 10.1016/j.jaap.2015.11.016.

[ref21] Bourdet A.; Esposito A.; Thiyagarajan S.; Delbreilh L.; Affouard F.; Knoop R. J. I.; Dargent E. Molecular Mobility in Amorphous Biobased Poly(Ethylene 2,5-Furandicarboxylate) and Poly(Ethylene 2,4-Furandicarboxylate). Macromolecules 2018, 51, 1937–1945. 10.1021/acs.macromol.8b00108.

[ref22] Miyagawa N.; Suzuki T.; Okano K.; Matsumoto T.; Nishino T.; Mori A. Synthesis of Furan Dimer-Based Polyamides with a High Melting Point. J. Polym. Sci., Part A: Polym. Chem. 2018, 56, 1516–1519. 10.1002/pola.29031.

[ref23] Miyagawa N.; Ogura T.; Okano K.; Matsumoto T.; Nishino T.; Mori A. Preparation of Furan Dimer-Based Biopolyester Showing High Melting Points. Chem. Lett. 2017, 46, 1535–1538. 10.1246/cl.170647.

[ref24] Kainulainen T. P.; Sirviö J. A.; Sethi J.; Hukka T. I.; Heiskanen J. P. UV-Blocking Synthetic Biopolymer from Biomass-Based Bifuran Diester and Ethylene Glycol. Macromolecules 2018, 51, 1822–1829. 10.1021/acs.macromol.7b02457. 30258254 PMC6150733

[ref25] Kainulainen T. P.; Hukka T. I.; Özeren H. D.; Sirviö J. A.; Hedenqvist M. S.; Heiskanen J. P. Utilizing Furfural-Based Bifuran Diester as Monomer and Comonomer for High-Performance Bioplastics: Properties of Poly(Butylene Furanoate), Poly(Butylene Bifuranoate), and Their Copolyesters. Biomacromolecules 2020, 21, 743–752. 10.1021/acs.biomac.9b01447. 31790208

[ref26] Ahmed A. M.; Kainulainen T. P.; Heiskanen J. P. Furfural-Based Modification of PET for UV-Blocking Copolymers with Decreased Oxygen Permeability. Ind. Eng. Chem. Res. 2021, 60, 7495–7504. 10.1021/acs.iecr.1c00629.

[ref27] Edling H. E.; Sun H.; Paschke E.; Schiraldi D. A.; Tanko J. M.; Paradzinsky M.; Turner S. R. High Barrier Biosourced Polyester from Dimethyl [2,2′-Bifuran]-5,5′-Dicarboxylate. Polymer 2020, 191, 122258 10.1016/j.polymer.2020.122258.

[ref28] Lotzs M.; Kandel K.; Salciccioli M.; Cohn S.; Galuska A. A.; Guzman J.; Turner S. R.; Edling H. E.; Paschke E. E. Bifuran-Modified Polyesters. WO 2020106511 A1, May 28, 2020.

[ref29] Lei Y.; Zhang S.; Shen G.; Zhu J.; Xue J.-W.; Chen Z.; Yin G. Feasible Synthesis of a Bifuran-Based Monomer for Polymer Synthesis from a Hemicellulose-Derived Platform. Ind. Eng. Chem. Res. 2020, 59, 19876–19883. 10.1021/acs.iecr.0c04203.

[ref30] Kainulainen T. P.; Erkkilä P.; Hukka T. I.; Sirviö J. A.; Heiskanen J. P. Application of Furan-Based Dicarboxylic Acids in Bio-Derived Dimethacrylate Resins. ACS Appl. Polym. Mater. 2020, 2, 3215–3225. 10.1021/acsapm.0c00367.

[ref31] Sun L.; Zhang Y.; Wang J.; Liu F.; Jia Z.; Liu X.; Zhu J. 2,5-Furandicarboxylic Acid as a Sustainable Alternative to Isophthalic Acid for Synthesis of Amorphous Poly(Ethylene Terephthalate) Copolyester with Enhanced Performance. J. Appl. Polym. Sci. 2019, 136, 47186–4. 10.1002/app.47186.

[ref32] Patel A. A.; Patel S. R. Polyschiff Bases-I. Syntheses and Properties of Polyschiff Bases from 5,5′-Thiobisfurfural. Eur. Polym. J. 1983, 19, 561–564. 10.1016/0014-3057(83)90177-5.

[ref33] Brahmbhatt D. I.; Patel H. S. Polychalcones Based on 5, 5’-Thio-Bis-2- Furancarboxaldehyde. Phosphorus, Sulfur Silicon Relat. Elem. 1992, 73, 57–62. 10.1080/10426509208034431.

[ref34] Farah S.; Kunduru K. R.; Basu A.; Domb A. J. Molecular Weight Determination of Polyethylene Terephthalate. In Poly(Ethylene Terephthalate) Based Blends, Composites and Nanocomposites; Visakh P. M., Liang M., Eds., 1st ed.; William Andrew, 2015; pp 143–165.

[ref35] Badovskaya L. A.; Poskonin V. V. Rearrangements and Tautomeric Transformations of Heterocyclic Compounds in Homogeneous Reaction Systems Furfural–H_2_O_2_–Solvent. Russ. J. Gen. Chem. 2018, 88, 1568–1579. 10.1134/S1070363218080030.

[ref36] Badovskaya L. A.; Poskonin V. V.; Povarova L. V. Synthesis of Functional Furan Derivatives by Oxidation of Furans and Formylfurans with Hydrogen Peroxide. Russ. Chem. Bull. 2017, 66, 593–599. 10.1007/s11172-017-1778-8.

[ref37] Travis B. R.; Sivakumar M.; Hollist G. O.; Borhan B. Facile Oxidation of Aldehydes to Acids and Esters with Oxone. Org. Lett. 2003, 5, 1031–1034. 10.1021/ol0340078. 12659566

[ref38] Grigg R.; Knight J. A.; Sargent M. V. Studies in Furan Chemistry. Part I. The Infrared Spectra of 2,5-Disubstituted Furans. J. Chem. Soc. 1965, 6057–6060. 10.1039/JR9650006057.

[ref39] Balani K.; Verma V.; Agarwal A.; Narayan R. Biosurfaces: A Materials Science and Engineering Perspective, 1st ed.; John Wiley & Sons: New Jersey, 2015.

[ref40] Stoclet G.; Gobius Du Sart G.; Yeniad B.; De Vos S.; Lefebvre J. M. Isothermal Crystallization and Structural Characterization of Poly(Ethylene-2,5-Furanoate). Polymer 2015, 72, 165–176. 10.1016/j.polymer.2015.07.014.

[ref41] Tsanaktsis V.; Vouvoudi E.; Papageorgiou G. Z.; Papageorgiou D. G.; Chrissafis K.; Bikiaris D. N. Thermal Degradation Kinetics and Decomposition Mechanism of Polyesters Based on 2,5-Furandicarboxylic Acid and Low Molecular Weight Aliphatic Diols. J. Anal. Appl. Pyrolysis 2015, 112, 369–378. 10.1016/j.jaap.2014.12.016.

[ref42] Guidotti G.; Soccio M.; García-Gutiérrez M.-C.; Gutiérrez-Fernández E.; Ezquerra T. A.; Siracusa V.; Munari A.; Lotti N. Evidence of a 2D-Ordered Structure in Biobased Poly(Pentamethylene Furanoate) Responsible for Its Outstanding Barrier and Mechanical Properties. ACS Sustain. Chem. Eng. 2019, 7, 17863–17871. 10.1021/acssuschemeng.9b04407.

[ref43] Terzopoulou Z.; Karakatsianopoulou E.; Kasmi N.; Tsanaktsis V.; Nikolaidis N.; Kostoglou M.; Papageorgiou G. Z.; Lambropoulou D. A.; Bikiaris D. N. Effect of Catalyst Type on Molecular Weight Increase and Coloration of Poly(Ethylene Furanoate) Biobased Polyester during Melt Polycondensation. Polym. Chem. 2017, 8, 6895–6908. 10.1039/c7py01171g.

[ref44] Gubbels E.; Jasinska-Walc L.; Noordover B. A. J.; Koning C. E. Linear and Branched Polyester Resins Based on Dimethyl-2,5-Furandicarboxylate for Coating Applications. Eur. Polym. J. 2013, 49, 3188–3198. 10.1016/j.eurpolymj.2013.06.019.

[ref45] Gruter G.-J. M.; Adrianus Dam M.; Adrianus Dam M. Accelerating Research into Bio-Based FDCA-Polyesters by Using Small Scale Parallel Film Reactors. Comb. Chem. High Throughput Screen. 2012, 15, 180–188. 10.2174/138620712798868374. 21902640

