# Supplementary material for: Renewable Furfural-Based Polyesters Bearing Sulfur-Bridged Difuran Moieties with High Oxygen Barrier Properties
Source: Biomacromolecules. 2022 Mar 23;23(4):1803–11. doi: 10.1021/acs.biomac.2c00097 (PMC9006217; doi:10.1021/acs.biomac.2c00097)
Supplement: Supplementary file 1 — bm2c00097_si_001.pdf [file bm2c00097_si_001.pdf]

Renewable Furfural-Based Polyesters Bearing Sulfur-Bridged Difuran Moieties with High Oxygen Barrier Properties

Asmaa M. Ahmed,[a] Tuomo P. Kainulainen,[a] Juho Antti Sirviö,[b] Juha P. Heiskanen*[a]

[a]Research Unit of Sustainable Chemistry, University of Oulu, P.O. Box 4300, FI-90014 Oulu, Finland

[b]Fibre and Particle Engineering Research Unit, University of Oulu, P.O. Box 4300, FI-90014 Oulu, Finland

*Corresponding Author: juha.heiskanen@oulu.fi

Table of Content

Figure S1: Characterization data of 5,5’-sulfanediyldi(furan-2-carbaldehyde) (**1**) S3

Figure S2: Characterization data of 5,5’-sulfanediyldi(furan-2-carboxylic acid) (**2**) S5

Figure S3: Characterization data of dimethyl 5,5’-sulfanediyldi(furan-2-carboxylate) (**3**). S7

Figure S4: PESF ^{1}H and ^{13}C NMR spectra S9

Figure S5: PPSF ^{1}H and ^{13}C NMR spectra S10

Figure S6: PBSF ^{1}H and ^{13}C NMR spectra S11

Figure S7: PPeSF ^{1}H and ^{13}C NMR spectra S12

Figure S8: FTIR spectra of polyesters PESF, PPSF, and PBSF S13

Figure S9: DSC thermograms of synthesized polyesters (1st heating-cooling scan) S14

Figure S10: DMA thermograms of melt-pressed polyesters films S15

Table S1: Storage modulus at 20 °C and (T_g) determined by DMA for the synthesized polyesters S15

Figure S1 (a): ^{1}H NMR spectrum (-1–10 ppm) of 5,5'-sulfanediyldi(furan-2-carbaldehyde) (**1**) in $CDCl_3$.

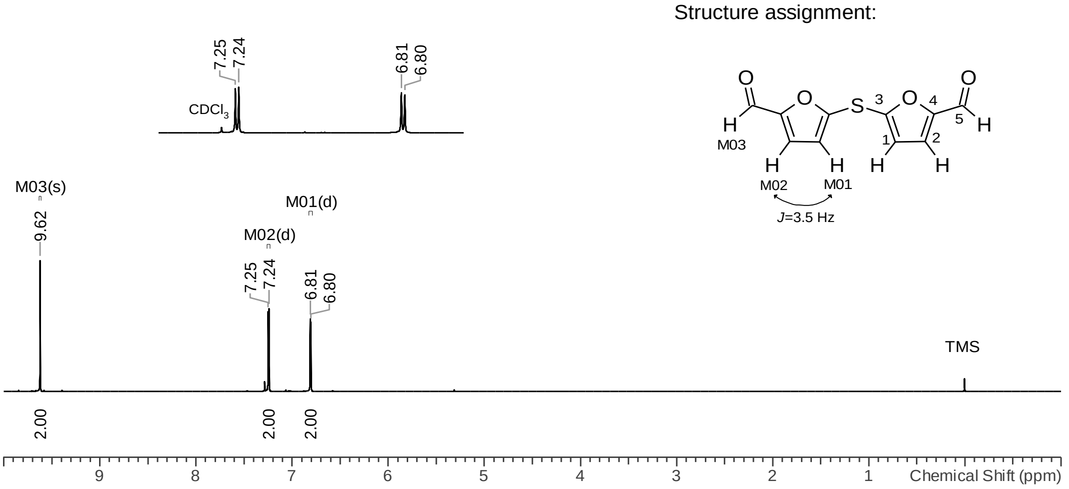

Figure S1 (b): ^{13}C NMR spectrum (-10–180 ppm) of 5,5'-sulfanediyldi(furan-2-carbaldehyde) (**1**) in $CDCl_3$.

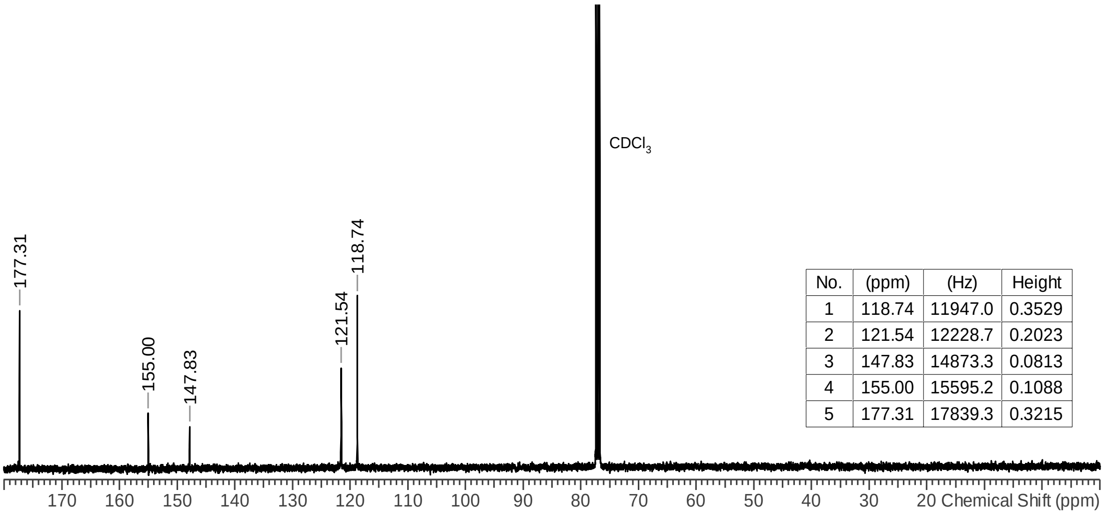

No.	(ppm)	(Hz)	Height
1	118.74	11947.0	0.3529
2	121.54	12228.7	0.2023
3	147.83	14873.3	0.0813
4	155.00	15595.2	0.1088
5	177.31	17839.3	0.3215

Figure S1 (c): HRMS spectrum of 5,5'-sulfanediyldi(furan-2-carbaldehyde) (**1**) for $C_{10}H_6O_4NaS$ $[M + Na]^+$.

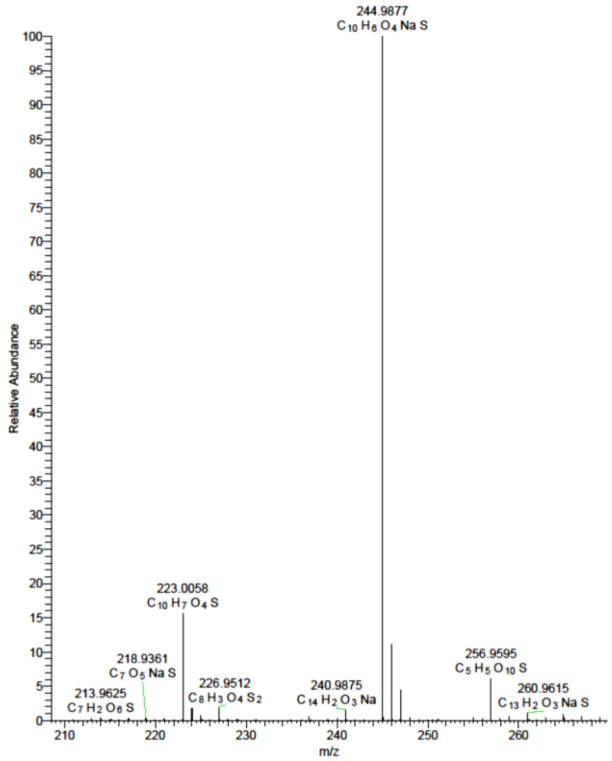

Figure S1 (d): DSC heating thermogram of 5,5'-sulfanediyldi(furan-2-carbaldehyde) (**1**).

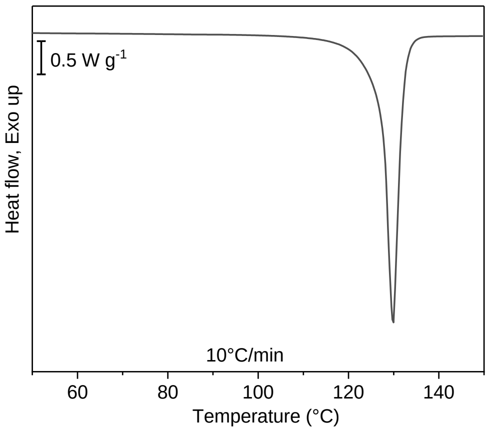

Figure S2 (a): ^{1}H NMR spectrum (-1–15 ppm) of 5,5'-sulfanediyldi(furan-2-carboxylic acid) (**2**) in DMSO-d_6.

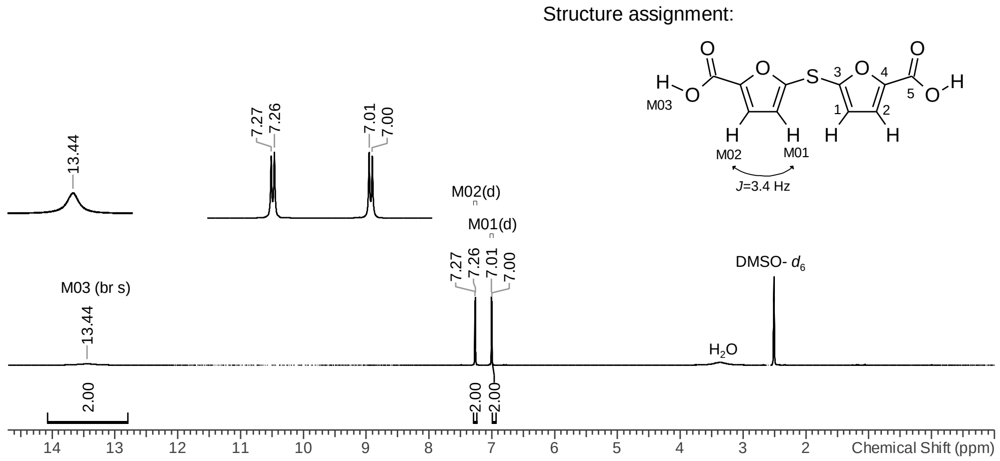

Figure S2 (b): ^{13}C NMR spectrum (-10–180 ppm) of 5,5'-sulfanediyldi(furan-2-carboxylic acid) (**2**) in DMSO-d_6.

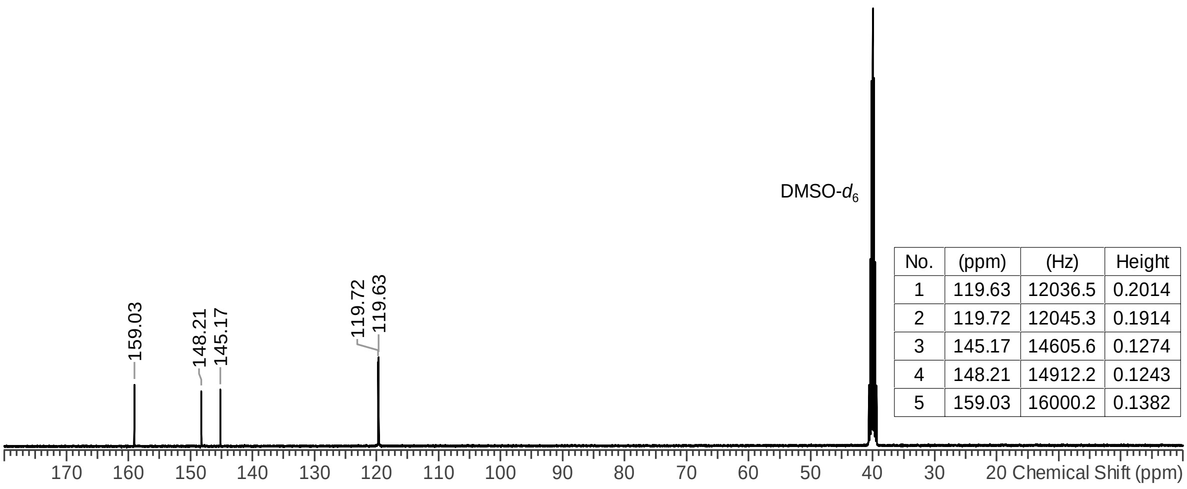

No.	(ppm)	(Hz)	Height
1	119.63	12036.5	0.2014
2	119.72	12045.3	0.1914
3	145.17	14605.6	0.1274
4	148.21	14912.2	0.1243
5	159.03	16000.2	0.1382

Figure S2 (c): FTIR spectrum (700–3600 cm^{-1}) of 5,5'-sulfanediyldi(furan-2-carboxylic acid) (**2**).

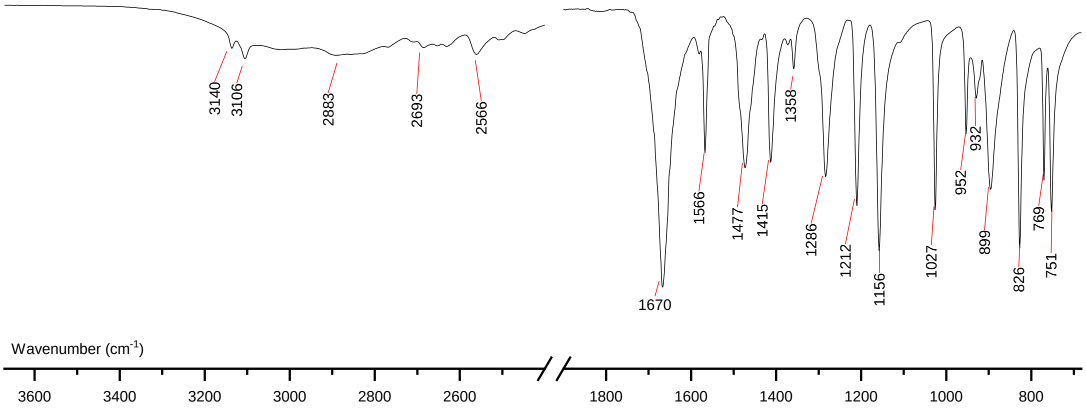

Figure S2 (d): HRMS spectrum of 5,5'-sulfanediyldi(furan-2-carboxylic acid) (**2**) for $C_{10}H_7O_6S$ $[M + H]^+$.

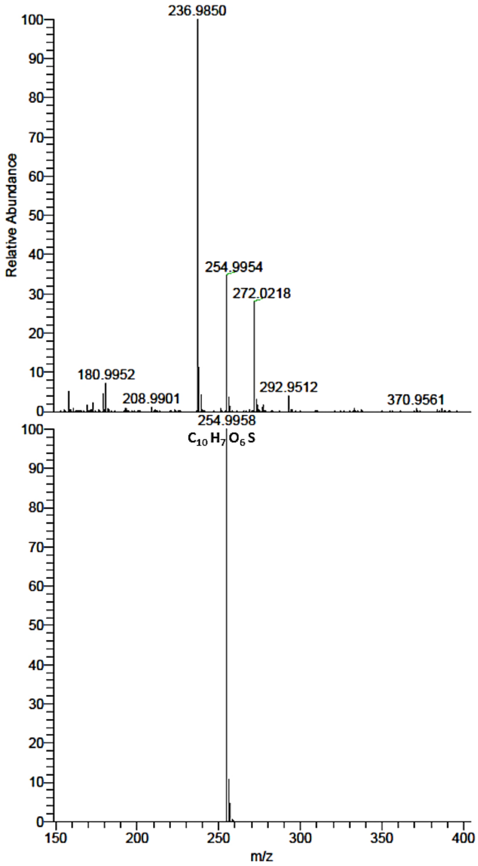

Figure S3 (a): ^{1}H NMR spectrum (-1–10 ppm) of dimethyl 5,5’-sulfanediyldi(furan-2-carboxylate) (**3**) in $CDCl_3$.

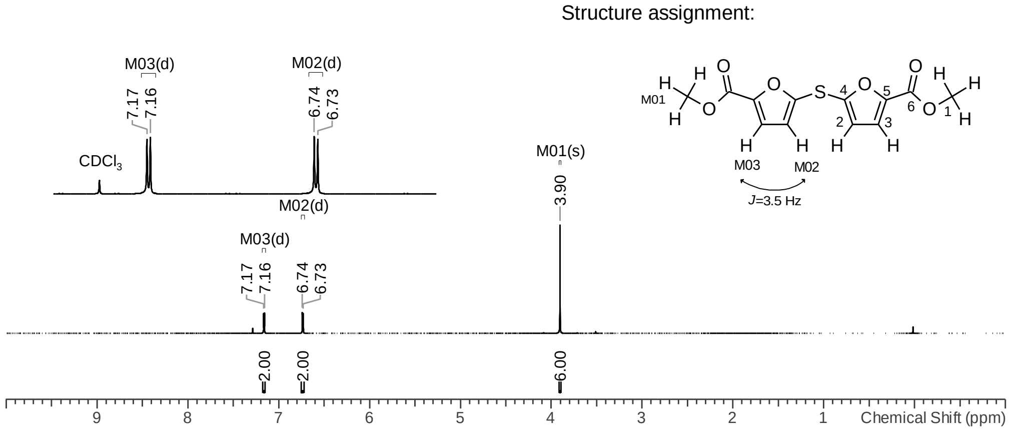

Figure S3 (b): ^{13}C NMR spectrum (-10–180 ppm) of dimethyl 5,5’-sulfanediyldi(furan-2-carboxylate) (**3**) in $CDCl_3$.

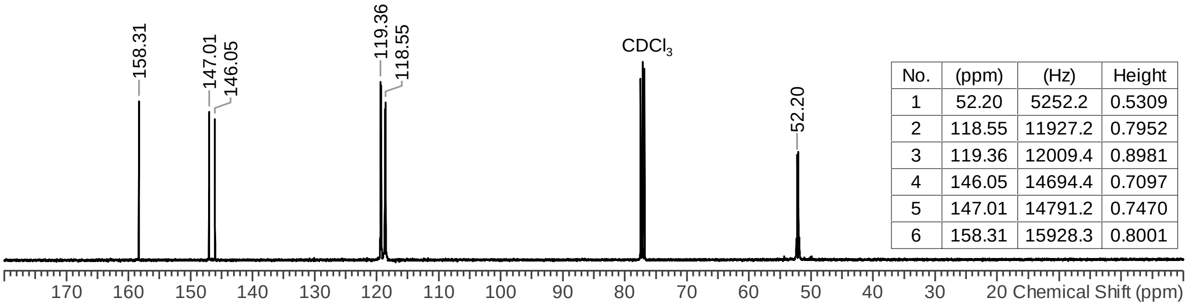

No.	(ppm)	(Hz)	Height
1	52.20	5252.2	0.5309
2	118.55	11927.2	0.7952
3	119.36	12009.4	0.8981
4	146.05	14694.4	0.7097
5	147.01	14791.2	0.7470
6	158.31	15928.3	0.8001

Figure S3 (c): FTIR spectrum (700–3600 cm^{-1}) of dimethyl 5,5’-sulfanediyldi(furan-2-carboxylate) (**3**).

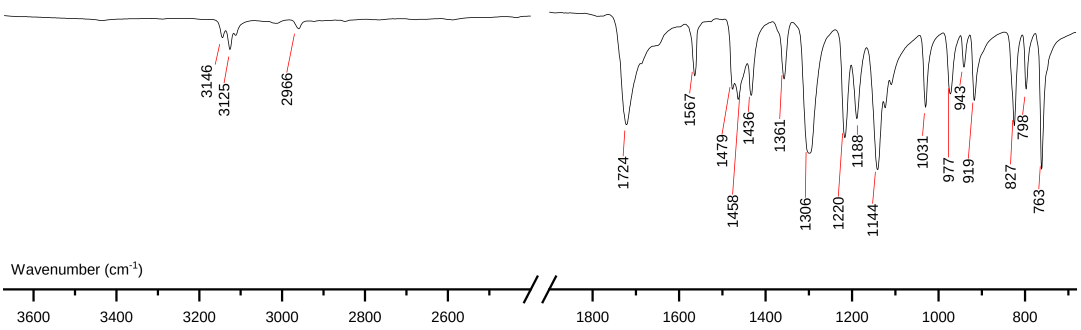

Figure S3 (d): HRMS spectrum of dimethyl 5,5'-sulfanediyldi(furan-2-carboxylate) (**3**) for $C_{12}H_{11}O_6S$ [M + H]$^+$.

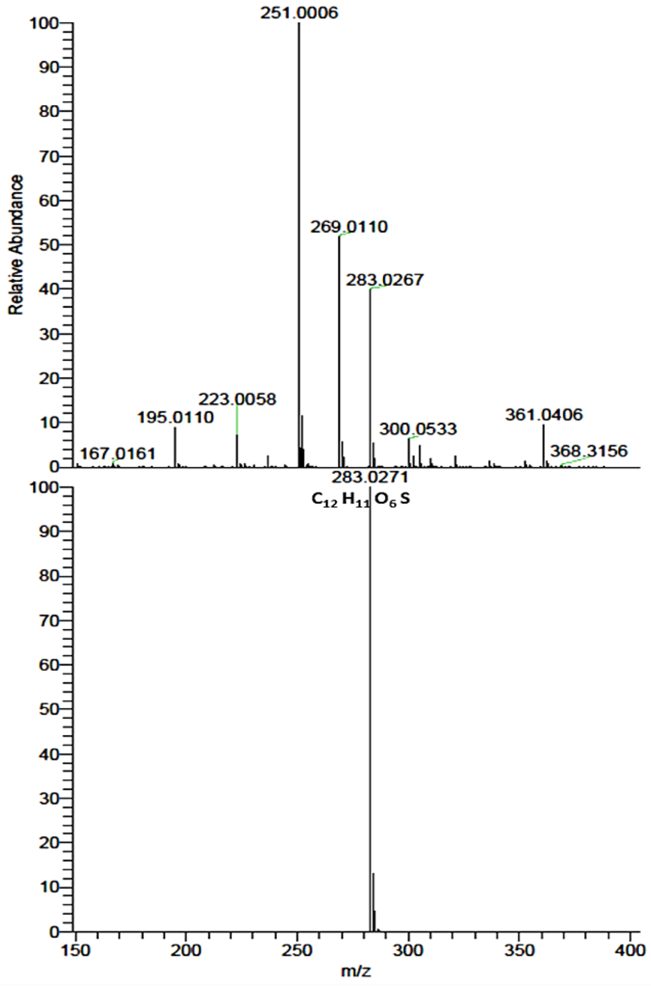

Figure S3 (e): DSC heating thermogram of dimethyl 5,5'-sulfanediyldi(furan-2-carboxylate) (**3**).

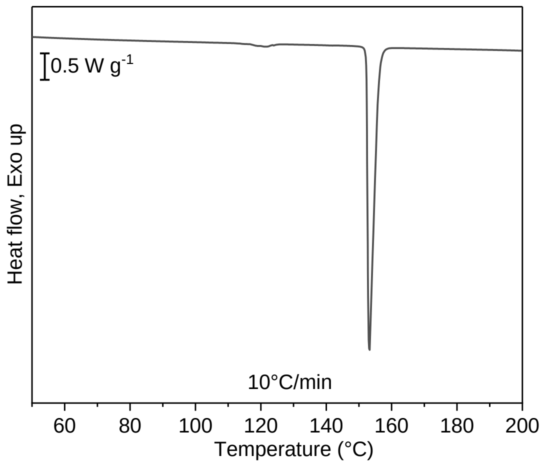

Figure S4 (a): ^{1}H NMR spectrum (-1–10 ppm) of PESF in $CF_3COOD:CDCl_3$ (1:3).

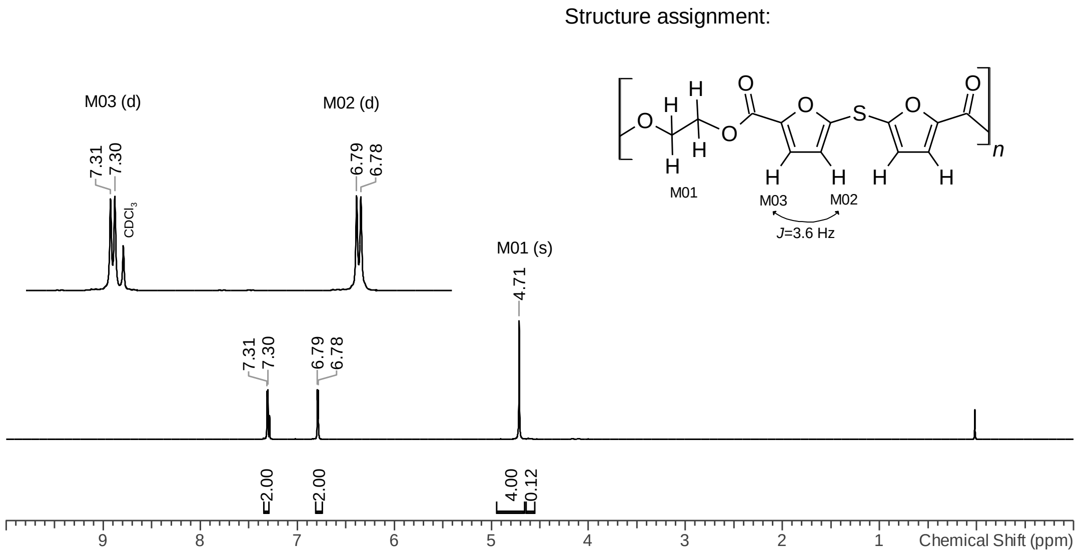

Figure S4 (b): ^{13}C NMR spectrum (-10–180 ppm) of PESF in CF_3COOD.

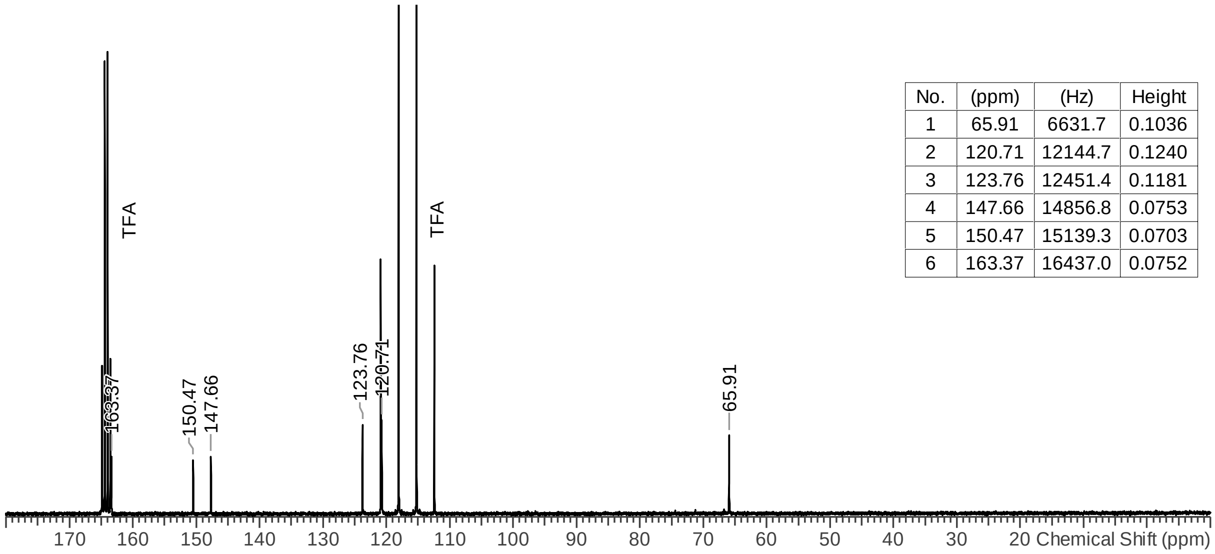

No.	(ppm)	(Hz)	Height
1	65.91	6631.7	0.1036
2	120.71	12144.7	0.1240
3	123.76	12451.4	0.1181
4	147.66	14856.8	0.0753
5	150.47	15139.3	0.0703
6	163.37	16437.0	0.0752

Figure S5 (a): ^{1}H NMR spectrum (-1–10 ppm) of PPSF in CF_3COOD:$CDCl_3$ (1:3).

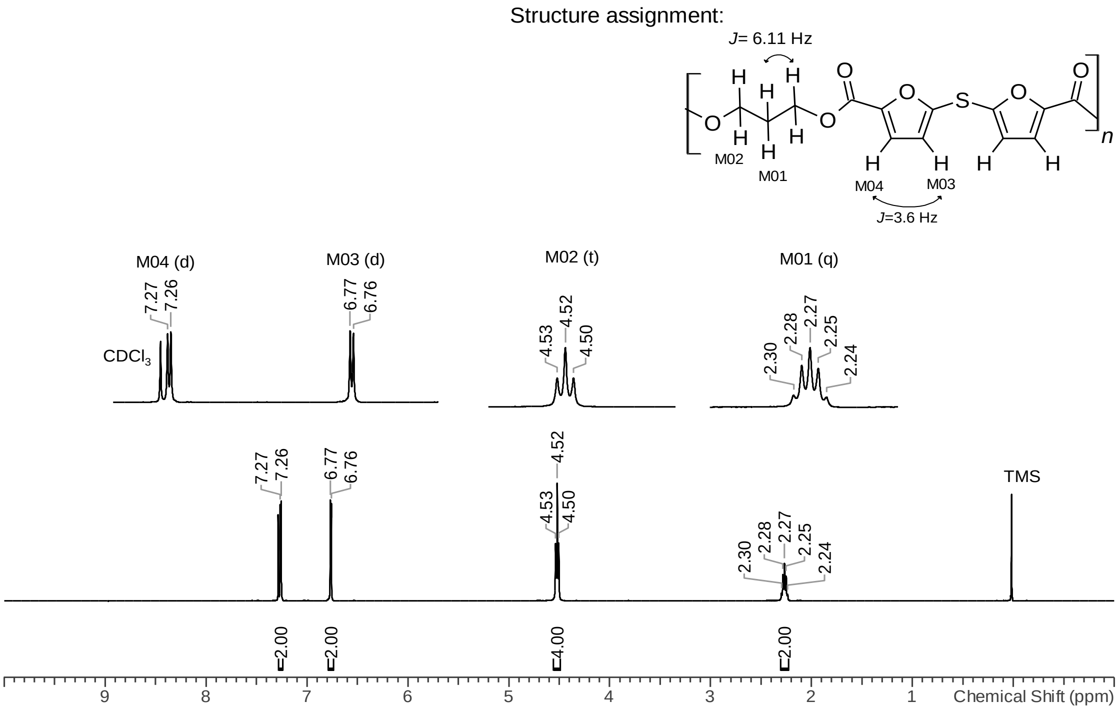

Figure S5 (b): ^{13}C NMR spectrum (-10–180 ppm) of PPSF in CF_3COOD.

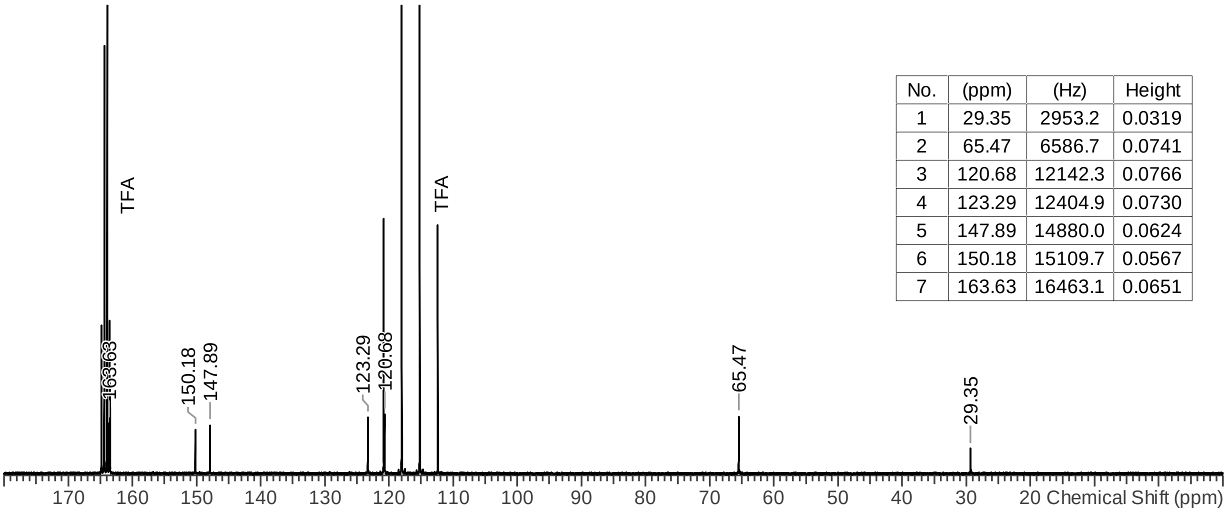

No.	(ppm)	(Hz)	Height
1	29.35	2953.2	0.0319
2	65.47	6586.7	0.0741
3	120.68	12142.3	0.0766
4	123.29	12404.9	0.0730
5	147.89	14880.0	0.0624
6	150.18	15109.7	0.0567
7	163.63	16463.1	0.0651

Figure S6 (a): ^{1}H NMR spectrum (-1–10 ppm) of PBSF in CF_3COOD:$CDCl_3$ (1:3).

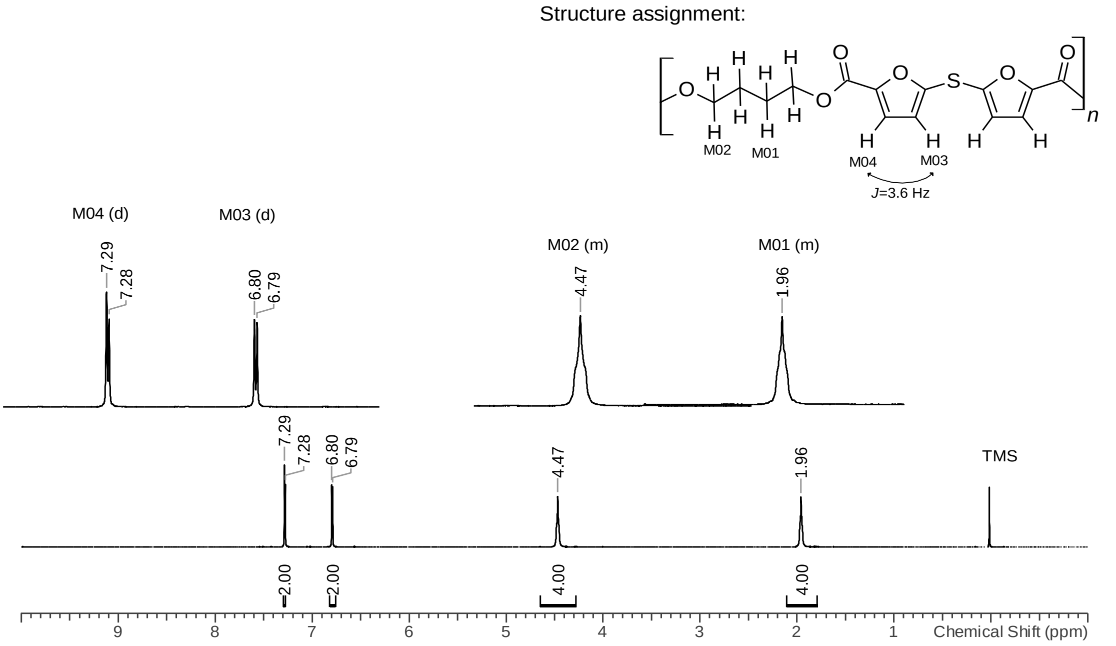

Figure S6 (b): ^{13}C NMR spectrum (-10–180 ppm) of PBSF in CF_3COOD.

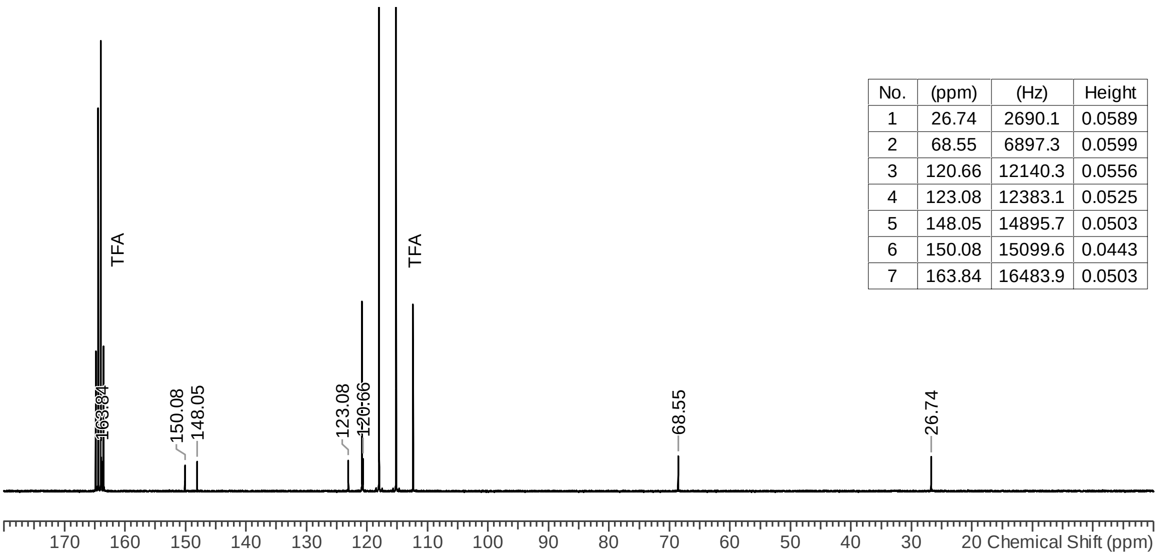

No.	(ppm)	(Hz)	Height
1	26.74	2690.1	0.0589
2	68.55	6897.3	0.0599
3	120.66	12140.3	0.0556
4	123.08	12383.1	0.0525
5	148.05	14895.7	0.0503
6	150.08	15099.6	0.0443
7	163.84	16483.9	0.0503

Figure S7 (a): ^{1}H NMR spectrum (-1–10 ppm) of PPeSF in $CF_3COOD:CDCl_3$ (1:3).

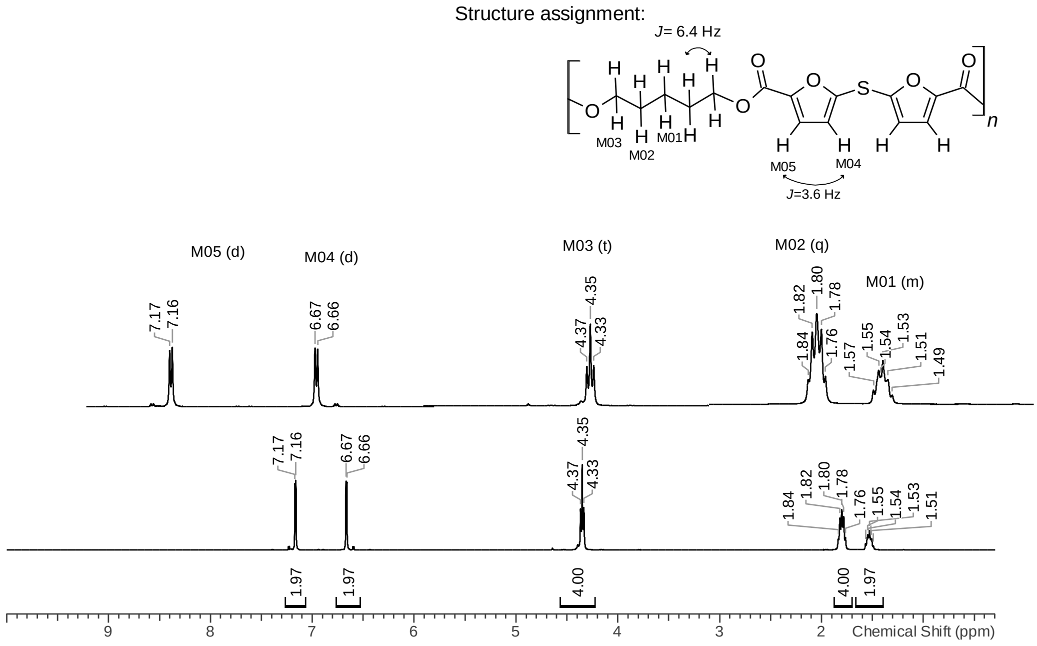

Figure S6 (b): ^{13}C NMR spectrum (-10–180 ppm) of PPeSF in CF_3COOD.

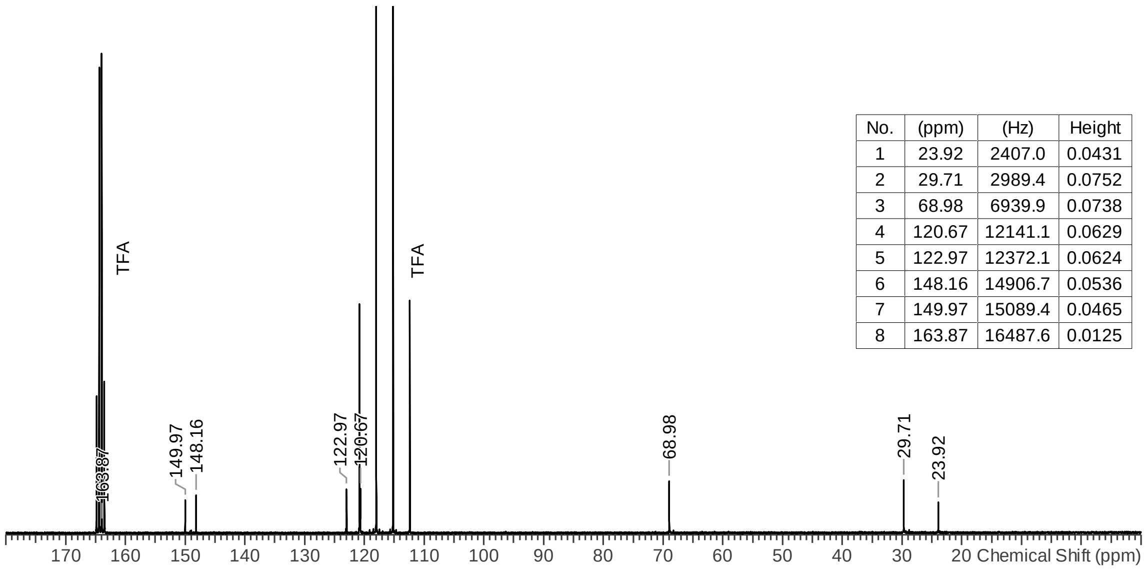

No.	(ppm)	(Hz)	Height
1	23.92	2407.0	0.0431
2	29.71	2989.4	0.0752
3	68.98	6939.9	0.0738
4	120.67	12141.1	0.0629
5	122.97	12372.1	0.0624
6	148.16	14906.7	0.0536
7	149.97	15089.4	0.0465
8	163.87	16487.6	0.0125

Figure S8 (a): FTIR spectrum (600–1900 cm^{-1}) of PESF, PPSF, and PBSF polyesters.

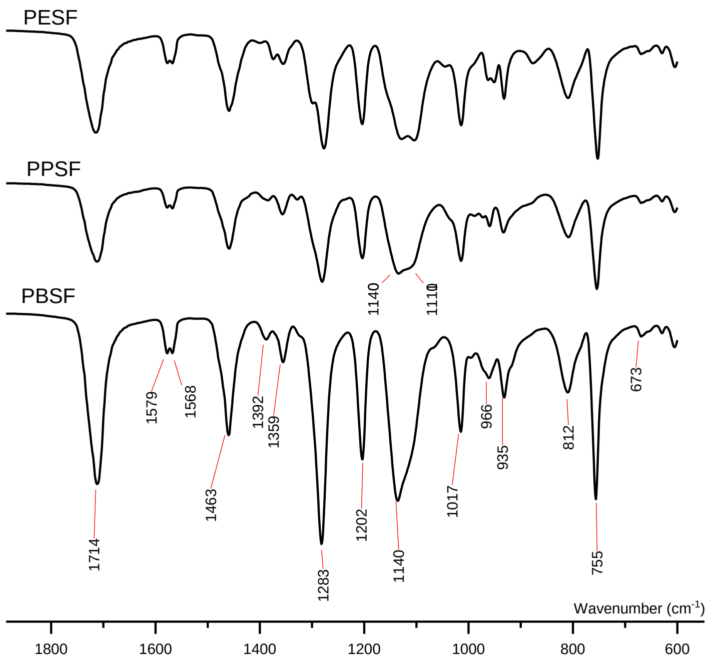

Figure S8 (b): FTIR amplified spectrum (2600–3400 cm^{-1}) of PESF, PPSF, and PBSF polyesters.

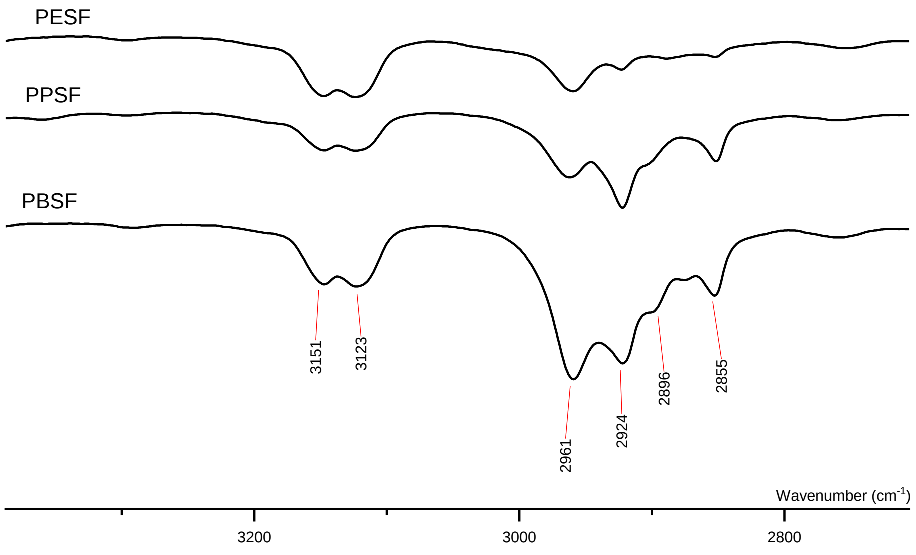

Figure S9: DSC thermograms of synthesized polyesters: (a) first heating scan and (b) first cooling scan.

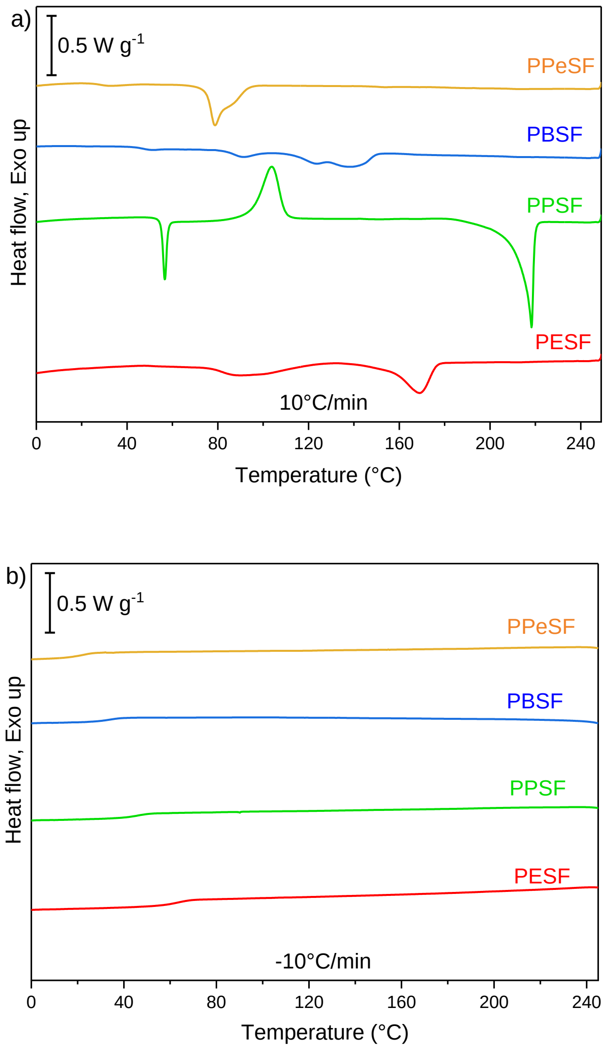

Figure S10: DMA thermograms of storage modulus *E*', loss modulus *E*'', and tan *δ* as a function of temperature for the melt-pressed polyester films: a) PESF, b) PPSF, c) PBSF, and d) PPeSF.

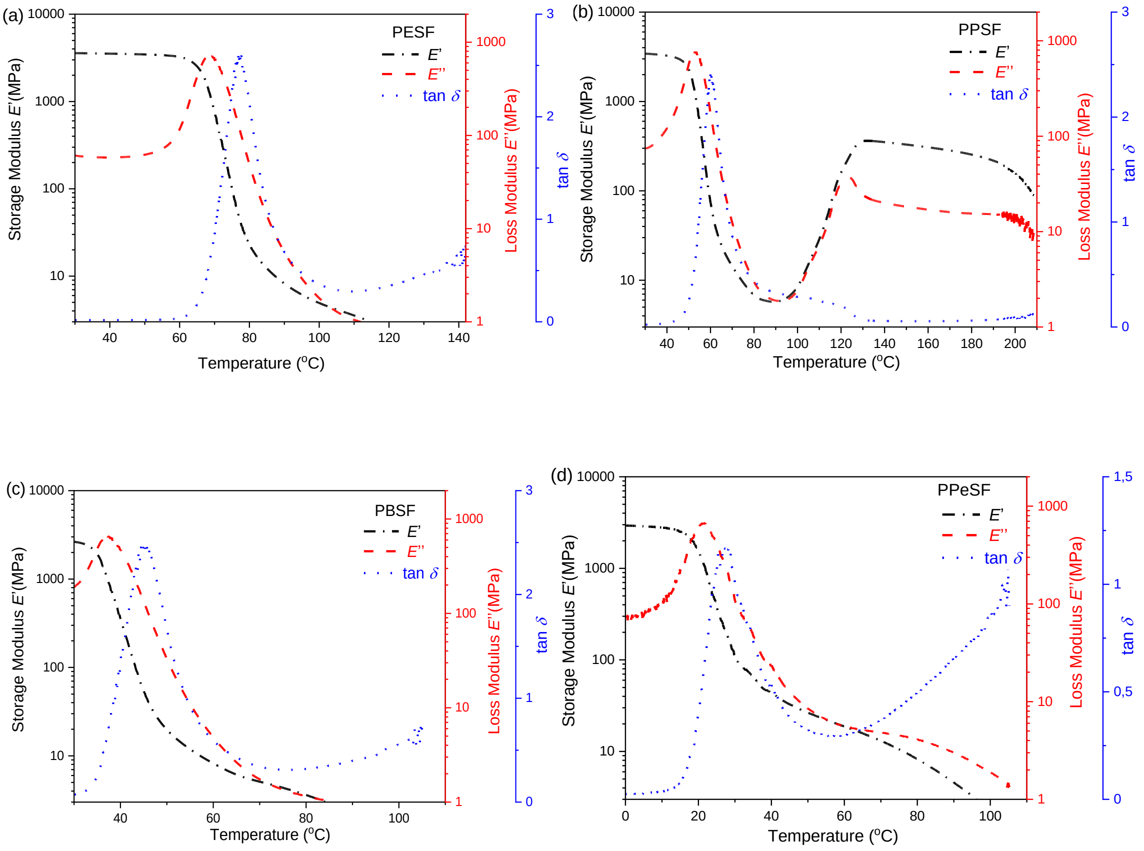

Table S1: Storage modulus at 20 °C and Glass transition temperatures (T_g) determined by DMA for the synthesized polyesters.

Polymer	Storage Modulus *E*´ at 20 °C (MPa)	T_g (°C)	
		Loss Modulus *E*´´	tan *δ*
PESF	3580	68	78
PPSF	3420	54	59
PBSF	2650	37	45
PPeSF	1290	22	27
